# FOXM1 Protects Against Myocardial Ischemia‐Reperfusion Injury in Rodent and Porcine Models by Suppressing MKRN1‐Dependent LKB1 Ubiquitination

**DOI:** 10.1002/advs.202508673

**Published:** 2025-06-23

**Authors:** Shuai Song, Xiaokai Zhang, Zihang Huang, Zhiqiang Pei, Linqi Zeng, Fengze Cai, Tongyao Wang, Mohan Li, Chenyan Liu, Yining Song, Jiahao Guo, Hao Lu, Xinyu Weng, Li Shen, Xiaochun Zhang, Xingxing Cai, Aijun Sun, Junbo Ge

**Affiliations:** ^1^ Department of Cardiology Zhongshan Hospital Fudan University Shanghai Institute of Cardiovascular Diseases Shanghai 200032 China; ^2^ Key Laboratory of Viral Heart Diseases National Health Commission Shanghai 200032 China; ^3^ Key Laboratory of Viral Heart Diseases Chinese Academy of Medical Sciences Shanghai 200032 China; ^4^ National Clinical Research Center for Interventional Medicine Shanghai 200032 China; ^5^ Department of Cardiology Xinhua Hospital Affiliated to Shanghai Jiaotong University School of Medicine Shanghai 200092 China; ^6^ Institutes of Biomedical Sciences Fudan University Shanghai 200032 China; ^7^ Xinxiang Medical University Henan 453003 China

**Keywords:** AMPK, cardiomyocytes, FOXM1, heart failure, mitochondria

## Abstract

Mitochondrial dysfunction is related to etiopathogenesis and progression of heart failure (HF). The underlying molecular mechanisms are not fully understood. Transcription factor FOXM1 plays an essential role in cardiovascular development. The present study explores its role in mitochondrial bioenergetics in postmitotic cardiomyocytes (CMs). FOXM1 is significantly upregulated in ischemic heart tissues from humans, mice, and pigs. CM‐specific *Foxm1*‐knockout mice exhibit dilated cardiomyopathy features associated with mitochondrial dysfunction. Transcriptomic and proteomic profiling of *Foxm1*‐knockout mice reveal robust, specific downregulation of gene programs important for mitochondrial energetics and homeostasis. Analysis of proteome and ubiquitinome data reveal that FOXM1 deficiency in CMs promotes LKB1 ubiquitination and impairs the AMPK signaling and energy metabolism pathways. Bioinformatics analysis identifies that E3 ligase MKRN1 promotes the K48‐linked ubiquitination of LKB1 on Lys146, which in turn, inhibits the AMPK signaling pathway and impairs energy homeostasis in mice with HF. CM‐specific *Mkrn1* knockout ameliorates cardiac dysfunction by rejuvenating the impaired mitochondrial bioenergetics induced by FOXM1 deficiency. FOXM1 overexpression preserves mitochondrial bioenergetics and protects against myocardial I/R injury in both rodent and porcine models. In conclusion, FOXM1 is actively involved in mitochondrial bioenergetics during HF. FOXM1 may be a potential promising therapeutic target for myocardial I/R injury and HF.

## Graphical abstract

FOXM1 maintains mitochondrial bioenergetic function by inhibiting MKRN1‐mediated ubiquitination of LKB1 in cardiomyocytes. Loss of FOXM1 in cardiomyocytes results in upregulation of MKRN1, which enhances LKB1 ubiquitination and disrupts AMPK signaling and energy metabolism pathways. Conversely, FOXM1 overexpression preserves mitochondrial bioenergetics and confers protection against cardiac ischemia–reperfusion injury in both murine and porcine models.

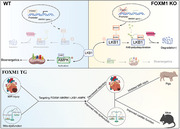

## 1 Introduction

Myocardial infarction (MI) is the leading cause of morbidity and mortality worldwide.^[^ ^1^ ^]^ Despite the implementation of revascularization and other therapy strategies, patients also develop heart failure (HF) due to the occurrence of ischemia‐reperfusion (I/R) injury.^[^ ^2^ ^]^ Therefore, major unmet clinical needs remain to explore the mechanisms underlying myocardial I/R injury and to develop novel therapeutic strategies for addressing post‐MI HF.

Myocardial I/R injury is characterized by a multifaceted process involving diverse mechanisms implicated in cardiac structural and functional damage.^[^ ^3^ ^]^ Mitochondria constitute more than 30% of the cardiomyocyte volume, providing most of the energy through electron transport chain (ETC) for sustaining the rhythmic beating of the heart.^[^ ^4^ ^]^ Functional defects in the mitochondrial ETC complexes I–V result in impaired mitochondrial energetics, cardiomyocytes death, and cardiac remodeling. The degree of mitochondrial damage plays a pivotal role in the progression of myocardial I/R injury toward HF.^[^ ^5^ ^]^ However, therapeutic interventions targeting mitochondrial energetics to alleviate post‐MI HF yield unsatisfactory outcomes.^[^ ^6^, ^7^ ^]^ Evidently, there exists a pressing necessity to deepen our comprehension of the intricate molecular and cellular mechanisms underlying reperfusion injury and to expand our investigations beyond the prevalent utilization of small rodent models in preclinical studies.

Multiple nuclear transcription factors (TFs) have been reported to play roles in the regulation of mitochondrial gene expression during I/R injury and HF.^[^ ^8^, ^9^, ^10^ ^]^ A previous study has shown that the FOX family TFs are associated with early HF in mice and advanced HF in humans.^[^ ^11^ ^]^ FOX protein M1 (FOXM1) shares homology with the other FOX family TFs due to its winged helix/forkhead DNA‐binding domain.^[^ ^12^ ^]^ FOXM1 has been demonstrated to play a key role in tissue repair following injury in the lung and liver.^[^ ^13^, ^14^ ^]^ During development, FOXM1 plays an important role in mediating smooth muscle cell (SMC) and cardiomyocyte (CM) proliferation. FOXM1 deficiency in SMCs or CMs induces arterial wall defects and ventricular hypoplasia, respectively, in mice.^[^ ^15^, ^16^, ^17^ ^]^ More recently, the roles of the FOX family proteins in various mitochondrial pathways have been reported. Markaisa et al. revealed that FOXM1 regulated mitochondrial homeostasis independent of nuclear transcription.^[^ ^18^ ^]^ However, the specific roles of FOXM1 in postnatal cardiac development and I/R injury, particularly in regard to energy homeostasis, remain elusive.

Here, we demonstrated elevated FOXM1 levels in cardiac samples from ischemic cardiomyopathy (ICM) patients as well as mice and pigs subjected to I/R injury. Conditional *Foxm1* knockout in CMs resulted in acute‐onset HF and rapid lethality due to impaired mitochondrial bioenergetics in mice. Further, FOXM1 overexpression attenuated I/R injury‐induced HF in C57BL/6 mice and Bama pigs via improving mitochondrial energy metabolism. Mechanistically, FOXM1‐regulated expression of E3 ligase makorin ring finger protein 1 (MKRN1) promoted the K48‐linked ubiquitination of liver kinase B1 (LKB1) on Lys146, which inhibited the AMP‐activated protein kinase (AMPK) signaling pathway and impaired energy homeostasis. Taken together, these data indicate that FOXM1 a mediator of mitochondrial homeostasis and the FOXM1/MKRN1/LKB1/AMPK axis might serve as a new therapeutic target of myocardial I/R injury and HF.

## 2 Results

### 2.1 *Foxm1* Expression Was Elevated in Ischemic Heart Tissues From Humans, Mice, and Pigs

**Figure 1:**
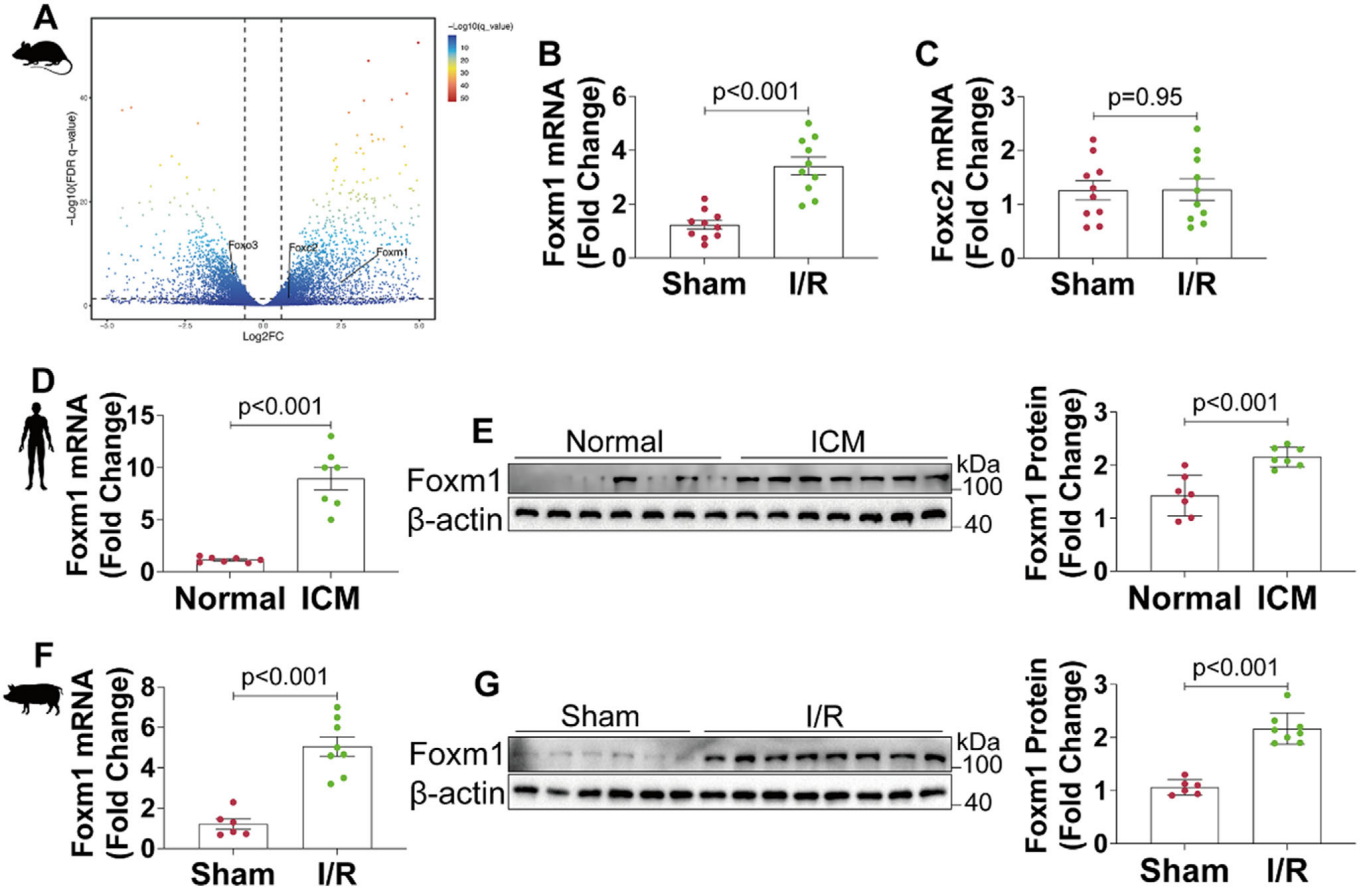
Foxm1 expression was elevated in ischemic heart tissues from humans, mice, and pigs. A) Volcano plot showing the gene expression of significantly altered FOX family members based on RNA‐seq data from heart samples of WT mice 24 h post‐I/R or Sham surgery. B,C) Quantitative real‐time polymerase chain reaction (qRT‐PCR) analyses of Foxm1 (B) and Foxc2 (C) mRNA levels in heart samples from WT mice 24 h post‐I/R or Sham surgery (*n* = 10). D) Quantitative real‐time polymerase chain reaction (qRT‐PCR) analyses of Foxm1 mRNA levels in heart samples from normal and ICM patients (*n* = 7). E) Western blot and quantification of FOXM1 protein levels in heart samples from normal and ICM patients (*n* = 7). F) Quantitative real‐time polymerase chain reaction (qRT‐PCR) analyses of Foxm1 mRNA levels in heart samples from Bama pigs 2 months post‐I/R or Sham surgery (*n* = 6 for Sham and *n* = 8 for I/R). G) Western blot and quantification of FOXM1 protein levels in heart samples from Bama pigs 2 months post‐I/R or Sham surgery (*n* = 6 for Sham and *n* = 8 for I/R). For all statistical plots, the data are presented as mean ± SD. (B,C) and (E–G) by two‐tailed unpaired Student's *t*‐test. (D) By Welch's *t*‐test.

To identify potential FOX factors involved in ischemic HF, we conducted an investigation into the differential expression of FOX factors among individuals diagnosed with ICM as well as a murine model of myocardial ischemia. Sridhar et al. have identified a subset of FOX factors responsible for the pathogenesis of human heart failure.^[^ ^11^ ^]^ However, our RNA‐seq analysis in a murine model of I/R injury revealed that only Foxm1 and Foxc2 exhibited upregulation in this context (false discovery rate *q*‐value < 0.05; fold change > 1.5) (**Figure** **1**A; Excel File S1, Supporting Information). Quantitative real time polymerase chain reaction (qRT‐PCR) confirmed that only Foxm1 maintained a consistent trend in both the heart samples of ICM patients and murine models of I/R injury (Figure 1B–D). Western blot further demonstrated increased FOXM1 protein levels in ICM samples (Figure 1E). In addition, we investigated whether these changes in FOXM1 were replicated in a porcine model of ischemic HF. Bama pigs underwent left anterior descending (LAD) coronary artery occlusion for 60 min, followed by reperfusion for 2 months. Both protein and mRNA levels of FOXM1 were significantly elevated in the hearts of pigs with myocardial I/R injury (Figure 1F,G). These findings provide novel insights into the involvement of the FOX family member FOXM1 in the pathogenesis of ischemic HF.

### 2.2 Inducible CM‐Specific Deletion of *Foxm1* in Adult Mice Exhibited Dilated Cardiomyopathy Features and High Lethality

**Figure 2:**
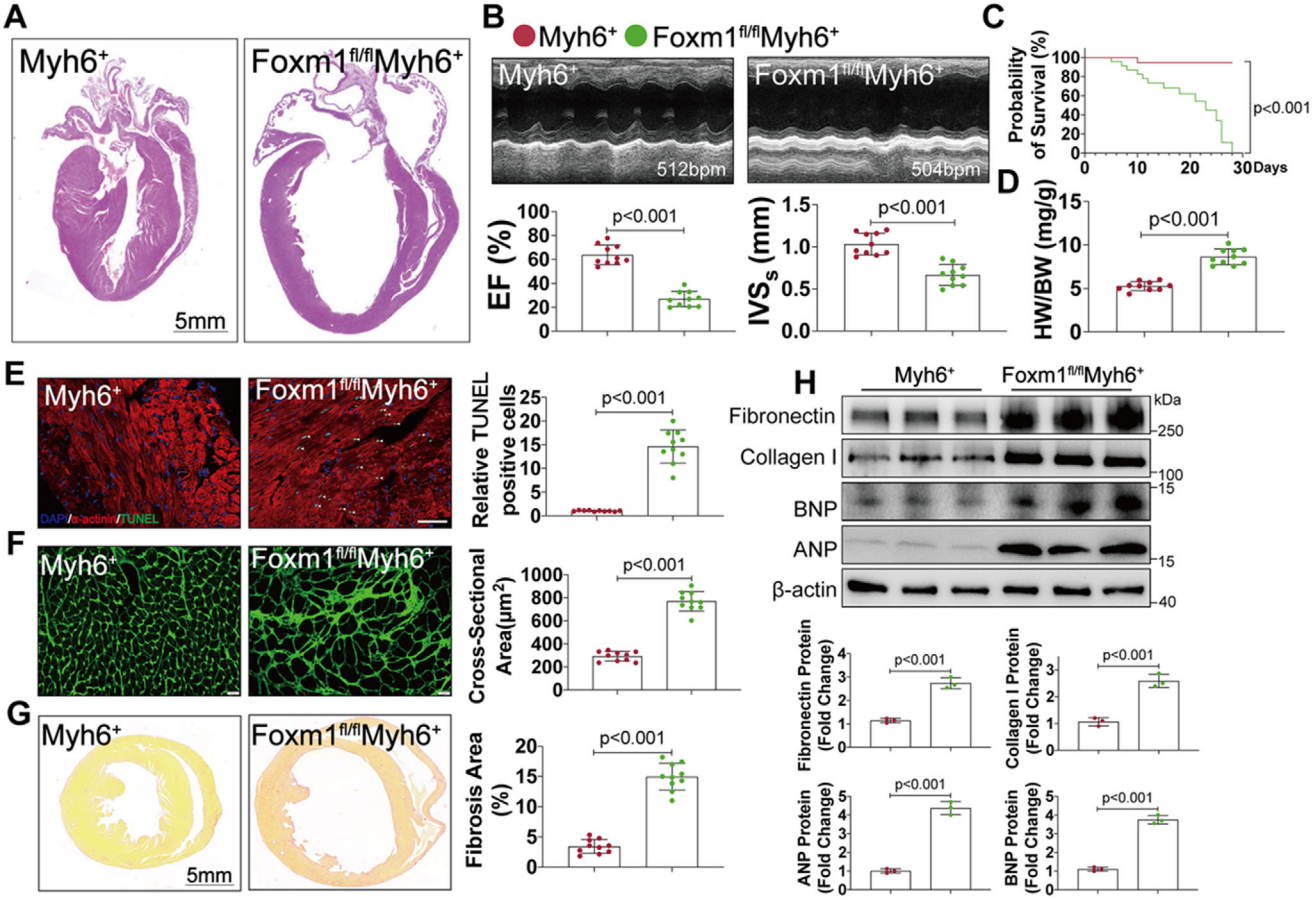
Cardiomyocytes‐specific FOXM1 deletion results in spontaneous dilated cardiomyopathy. A) Heart sections from *Myh6^+^* and *Foxm1^fl/fl^Myh6^+^* mice were stained with hematoxylin and eosin to show whole‐heart gross images (*n* = 10; scale bar = 5 mm). B) M‐mode echocardiography and relative echocardiographic parameters of *Myh6^+^* and *Foxm1^fl/fl^Myh6^+^* mice at 14 days after the first tamoxifen injection (*n* = 10). C) Survival rate of *Myh6* ^+^ and *Foxm1^fl/fl^Myh6^+^* mice was estimated by Kaplan–Meier method. D) The ratios of HW to BW of *Myh6^+^* and *Foxm1^fl/fl^Myh6^+^* mice at 14 days after the first tamoxifen injection (*n* = 10 per group). E) Heart sections from *Myh6* ^+^ and *Foxm1^fl/fl^Myh6^+^* mice were stained with TUNEL staining to detect cardiomyocytes apoptosis (*n* = 10; scale bar = 20 µm). F) Heart sections from *Myh6^+^* and *Foxm1^fl/fl^Myh6^+^* mice were stained with WGA to demarcate the cell boundaries (*n* = 10; scale bar = 20 µm). G) Heart sections from *Myh6^+^* and *Foxm1^fl/fl^Myh6^+^* mice were stained with picrosirius red to visualize collagen deposition (*n* = 10; scale bar = 5 mm). H) Western blot and quantification of Fibronectin, Collagen I, Anp, and Bnp protein levels in heart samples from *Myh6* ^+^ and *Foxm1^fl/fl^Myh6^+^* mice (*n* = 3). For all statistical plots, the data are presented as mean ± SD. (B,D,F–H) By two‐tailed unpaired Student's *t*‐test. (C) By log‐rank test. (E) By Welch's *t*‐test. LVEF, left ventricular ejection fraction; IVS, interventricular septum; and HW/BW, ratio of heart weight to body weight.

To further investigate the role of FOXM1 in heart, CM‐specific *Foxm1*‐knockout (*Foxm1^fl/fl^Myh6* ^+^) mice were generated by crossing *Foxm1*‐floxed (*Foxm1^fl/fl^*) mice with *Myh6*‐MerCreMer mice; thus, allowing for the tamoxifen (TAM)‐inducible, CM‐specific knockout of *Foxm1* (Figure S1A, Supporting Information). The genotypes of the mice were determined by PCR (Figure S1B, Supporting Information). After 5 days of 25 mg kg^−1^ TAM administration, FOXM1 expression was decreased in heart tissues of *Foxm1^fl/fl^Myh6* ^+^ mice compared with the control *Myh6^+^* mice (Figure S1C,D, Supporting Information). qRT‐PCR and Western blot also confirmed the specific deletion of FOXM1 in CMs (Figure S1E,F, Supporting Information). Echocardiography and histological analyses were performed on surviving *Foxm1^fl/fl^Myh6^+^* mice at 14 days after TAM injection. Histological analysis revealed HF features in these mice, indicated by enlarged ventricular chambers and thinning of the ventricular wall (**Figure** **2**A). Echocardiographic assessment showed a significant reduction in both the left ventricular ejection fraction (LVEF) and interventricular septum (IVS) in *Foxm1^fl/fl^Myh6^+^* mice compared with the *Myh6^+^* controls (Figure 2B). Detailed cardiac ultrasound data are provided in Table S5, Supporting Information. Notably, the *Foxm1^fl/fl^Myh6* ^+^ mice began to succumb after 5 days of TAM injection, with no survivors beyond 30 days (Figure 2C). In addition, Foxm1 knockout hearts were markedly larger and exhibited increased heart weight (Figure 2D). TUNEL staining and wheat germ agglutinin (WGA) staining demonstrated elevated CMs apoptosis and increased myocardial cross‐sectional area (CSA) in *Foxm1^fl/fl^Myh6* ^+^ mice compared with control hearts, suggesting a loss of cardiomyocytes and compensatory hypertrophy in the remaining cardiomyocytes (Figure 2E,F). Picrosirius red staining revealed excessive collagen deposition in *Foxm1^fl/fl^Myh6* ^+^ hearts, indicating increased cardiac fibrosis, a hallmark of pathological cardiac remodeling (Figure 2G). At the molecular level, FOXM1 deficiency in CMs significantly upregulated fibronectin, collagen I, atrial natriuretic peptide (ANP), and brain natriuretic peptide (BNP) in left ventricular heart tissues (Figure 2H). Altogether, these data suggest that FOXM1 plays an essential role in maintaining cardiac homeostasis and that CM‐specific *Foxm1* knockout leads to rapid lethality due to HF.

### 2.3 FOXM1 Expression in CMs is Indispensable for Maintaining Mitochondrial Bioenergetics

To investigate the underlying mechanism of FOXM1 deficiency‐driven HF, comprehensive RNA‐seq analyses were performed in left ventricular samples from *Foxm1^fl/fl^Myh6* ^+^ mice and *Myh6* ^+^ mice 14 days post TAM injection. The analysis revealed 1457 and 1171 significantly up‐ and downregulated genes (false discovery rate *q*‐value <0.05; fold change >1.5 or fold change < 1/1.5) in the samples from *Foxm1^fl/fl^Myh6* ^+^ mice compared to *Myh6* ^+^ mice (Figure S2A and Excel File S2, Supporting Information). Comparative gene ontology (GO) analysis indicated that the upregulated genes were primarily enriched in ECM organization (Figure S2B, Supporting Information); while, the downregulated genes were notably associated with mitochondrial metabolic pathways (Figure S2C, Supporting Information). Hierarchical clustering analysis demonstrated that the knockout of Foxm1 led to the downregulation of mitochondrial ETC complex proteins and the upregulation of ECM deposition molecules (Figure S2D, Supporting Information). To verify these results, we assessed the mRNA and protein expression levels of the ETC complex subunits as well as those related to ECM organization in vivo. As expected, the expression of the genes and proteins associated with the ETC complex subunits were downregulated in the *Foxm1^fl/fl^Myh6* ^+^ samples compared with the *Myh6* ^+^ samples, whereas those associated with ECM organization were markedly upregulated (Figure S2E–G, Supporting Information). In addition, reduced oxidative phosphorylation was positively associated with compromised mitochondrial bioenergetics. Transmission electron microscopy (TEM) revealed that FOXM1 deficiency in CMs resulted in abnormal mitochondrial morphology and reduced mitochondrial cristae density (Figure S2H, Supporting Information). These findings suggest a critical and indispensable role of FOXM1 in preserving normal cardiac mitochondrial function, which is analogous to the phenotypic observations in the *Foxm1^fl/fl^Myh6* ^+^ mice.

**Figure 3:**
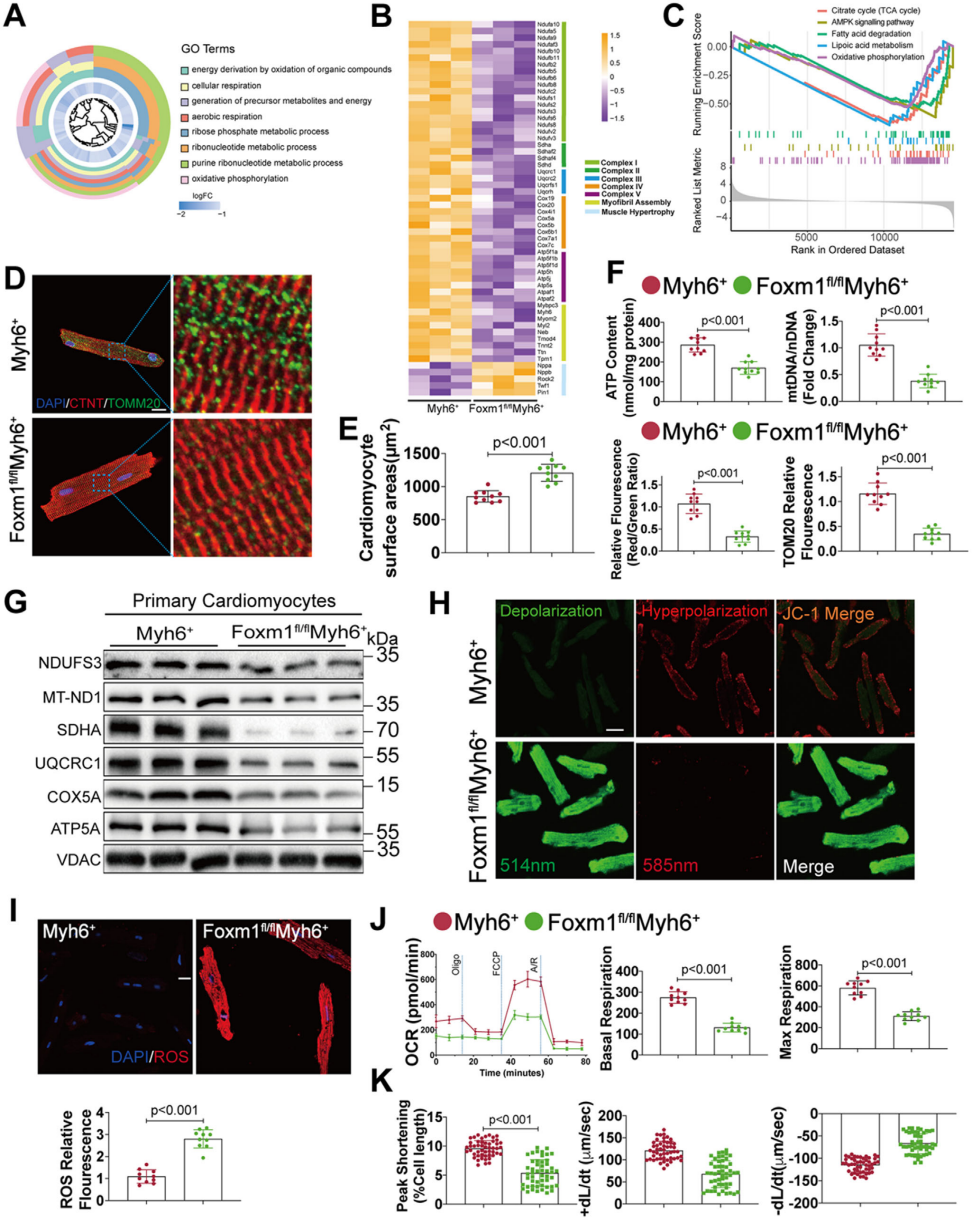
FOXM1 deficiency resulted in mitochondrial dysfunction in cardiomyocytes. A) GO term enrichment analysis of downregulated proteins in cardiomyocytes isolated from *Myh6* ^+^ and *Foxm1^fl/fl^Myh6^+^* mice based on MS data showing the top eight biological process. B) Heatmaps of mitochondrial ETC complex proteins in cardiomyocytes isolated from *Myh6* ^+^ and *Foxm1^fl/fl^Myh6^+^* mice based on MS data. C) GSEA analysis of differentially expressed proteins in cardiomyocytes isolated from *Myh6* ^+^ and *Foxm1^fl/fl^Myh6^+^* mice based on MS data. D) Immunofluorescence revealed mitochondrial morphology in cardiomyocytes isolated from *Myh6* ^+^ and *Foxm1^fl/fl^Myh6^+^* mice (*n* = 10; scale bar = 10 µm in left panel and 1 µm in right panel). E) Cardiomyocyte surface areas of cardiomyocytes isolated from *Myh6* ^+^ and *Foxm1^fl/fl^Myh6^+^* mice (*n* = 10). F) ATP levels and the ratio of mitochondrial DNA (mtDNA) to nuclear DNA (nDNA) in homogenates of isolated cardiomyocytes in *Myh6* ^+^ and *Foxm1^fl/fl^Myh6^+^* mice (*n* = 10). G) Western blot of mitochondrial complex proteins in cardiomyocytes isolated from *Myh6* ^+^ and *Foxm1^fl/fl^Myh6^+^* mice (*n* = 3). H) Representative confocal images of mitochondrial transmembrane potential (Δ*ψm*) labeled by JC‐1 monomer (green, marking depolarization) and JC‐1 aggregate (red, marking hyperpolarization) in cardiomyocytes isolated from *Myh6* ^+^ and *Foxm1^fl/fl^Myh6^+^* mice (*n* = 10; scale bar = 20 µm). I) Representative micrograph and quantitative analysis of intracellular reactive oxygen species (ROS) in cardiomyocytes isolated from *Myh6* ^+^ and *Foxm1^fl/fl^Myh6^+^* mice (*n* = 10). J) Seahorse real‐time traces and averaged data for mitochondrial oxygen consumption rate (OCR) were measured in cardiomyocytes isolated from *Myh6* ^+^ and *Foxm1^fl/fl^Myh6^+^* mice using Seahorse XFe24 Analyzer (*n* = 10). K) Contractile properties of cardiomyocytes isolated from *Myh6* ^+^ and *Foxm1^fl/fl^Myh6^+^* mice (*n* = 50). +dL/dt, maximal velocity of shortening; −dL/dt, maximal velocity of relengthening. For all statistical plots, the data are presented as mean ± SD. (E,F) and (I,J) By two‐tailed unpaired Student's *t*‐test. (K) By Welch's *t*‐test.

Although the bioinformatics analysis indicated impaired mitochondrial function, the results may have been confounded by the various types of cells in the heart. Therefore, we performed MS in adult CMs isolated from *Myh6^+^* and *Foxm1^fl/fl^Myh6^+^* mice after TAM injection. The analysis revealed 515 significantly increased and 462 decreased proteins in the isolated CMs from *Foxm1^fl/fl^Myh6* ^+^ mice in comparison to *Myh6* ^+^ mice (Excel File S3, Supporting Information). GO analysis revealed that the proteins that were decreased in response to *Foxm1* knockout in CMs were associated with the energy derivation, cellular respiration, and oxidative phosphorylation (**Figure** **3**A). In line with the tissue results, many of these proteins were associated with the mitochondrial respiratory ETC and myofibril assembly (Figure 3B). Gene set enrichment analysis (GSEA) further revealed that the top five pathways affected by *Foxm1* knockout were all associated with mitochondrial energy metabolism (Figure 3C). Immunofluorescence analysis revealed fewer mitochondria and larger cardiomyocyte surface areas in the *Foxm1^fl/fl^Myh6* ^+^ CMs than the control CMs (Figure 3D,E). Further, the cellular ATP levels and mitochondrial DNA (mtDNA) content were also lower in the *Foxm1^fl/fl^Myh6* ^+^ CMs compared to *Myh6* ^+^ CMs (Figure 3F). Western blot confirmed that proteins associated with the ETC were decreased in response to *Foxm1* knockout (Figure 3G). As a result, the *Foxm1^fl/fl^Myh6* ^+^ CMs exhibited increased mitochondrial depolarization and ROS level (Figure 3H,I). Consistently, seahorse analysis demonstrated that *Foxm1* knockout in CMs dramatically decreased the mitochondrial O_2_ consumption rate and contractile function (Figure 3J,K). Collectively, these data suggest that FOXM1 deficiency in CMs impairs mitochondrial function and energy metabolism.

### 2.4 FOXM1‐Regulated Energy Metabolism Requires Activation of the LKB1‐AMPK Signaling Pathway

**Figure 4:**
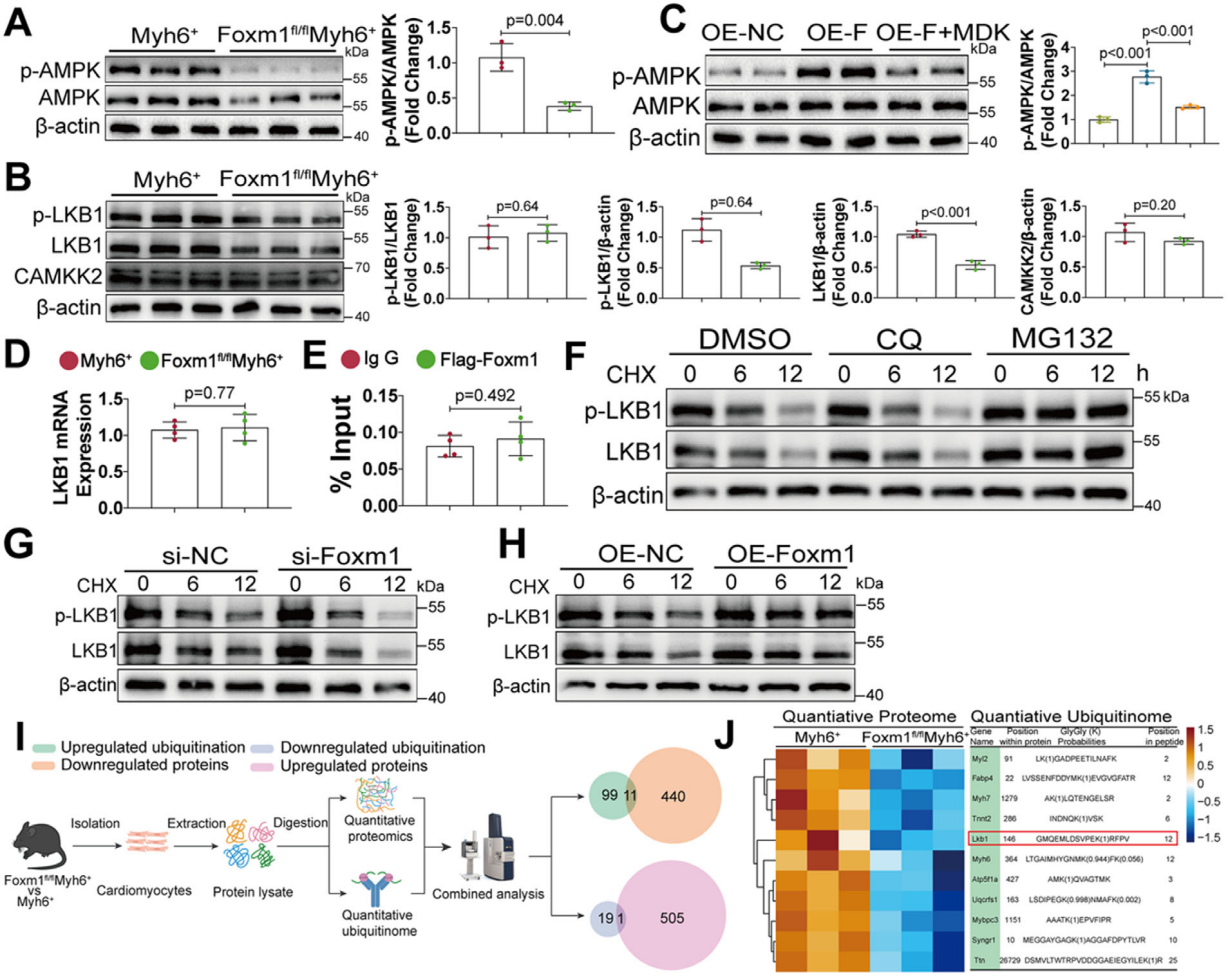
FOXM1 deficiency induces the ubiquitin‐dependent degradation of LKB1 to downregulate AMPK signaling. A) Western blot and quantification of p‐AMPK and AMPK protein levels in heart samples from *Myh6* ^+^ and *Foxm1^fl/fl^Myh6^+^* mice (*n* = 3). B) Western blot and quantification of p‐LKB1, LKB1, and CAMKK2 protein levels in heart samples from *Myh6* ^+^ and *Foxm1^fl/fl^Myh6^+^* mice (*n* = 3). C) Western blot and quantification of p‐AMPK and AMPK protein levels in cultured NVCMs transfected with OE‐*Foxm1* or OE‐NC follow treatment of LKB1 inhibitor MDK‐2275 (760 nm) (*n* = 3). D) qRT‐PCR analyses of LKB1 mRNA levels in heart samples of *Myh6* ^+^ and *Foxm1^fl/fl^Myh6^+^* mice (*n* = 4). E) ChIP‐qPCR analysis detected the enrichment of FOXM1 to *Lkb1* promoter region in HEK293T cells transfected with Flag‐Foxm1 plasmid (*n* = 4). F) Western blot and quantification of p‐LKB1 and LKB1 protein levels in NVCMs cultured with dimethylsulfoxide (DMSO), chloroquine (CQ, 50 µm), or MG132 (50 µm) with cycloheximide (CHX, 50 µm) for the indicated durations (*n* = 3). G) Western blot of p‐LKB1 and LKB1 in NVCMs transfected with si‐NC or si‐Foxm1 before treatment of cycloheximide (CHX, 50 µm) for the indicated durations (*n* = 3). H) Western blot of p‐LKB1 and LKB1 in NVCMs infected with adenovirus expressing *Foxm1* or NC before treatment of cycloheximide (CHX, 50 µm) for the indicated durations (*n* = 3). I) Schematic diagram showing the proteome and ubiquitinome‐based experimental design using cardiomyocytes isolated from *Myh6^+^* and *Foxm1^fl/fl^Myh6^+^* mice. J) Heatmap showing combined analysis of quantitative proteome and ubiquitinome results. For all statistical plots, the data are presented as mean ± SD. (A,B,D,E) By two‐tailed unpaired Student's *t*‐test. (C) By one‐way ANOVA with Bonferroni multiple comparison test.

Next, we aimed to investigate the potential mechanisms underlying the mitochondrial dysfunction induced by FOXM1 deficiency. Among the top five pathways identified by GSEA analysis, AMPK activation promoted mitochondrial biogenesis and oxidative phosphorylation, thereby exerting a regulatory influence over the other four pathways (Figure 3C). Consistent with the bioinformatics findings, Western blot analysis showed that phosphorylated AMPK was downregulated in response to FOXM1 deficiency (**Figure** **4**A). To evaluate whether AMPK inactivation was responsible for the mitochondrial dysfunction of *Foxm1* knockout CMs, we investigated the impact of AMPK activation in CMs isolated from *Foxm1^fl/fl^Myh6* ^+^ mice. A769662‐induced AMPK activation significantly mitigated the decline in oxygen consumption rate (OCR) associated with the mitochondrial electron transport chain (ETC) complex and reduced mitochondrial depolarization in *Foxm1* knockout CMs (Figure S3A,B, Supporting Information). These findings collectively suggest that FOXM1 may modulate mitochondrial function via the AMPK signaling pathway.

To further delineate the relationship between FOXM1 deficiency and AMPK inactivation, we investigated the known upstream kinases responsible for AMPK activation. The activation of AMPK predominantly occurs through phosphorylation of the Thr172 activation site located within its α‐catalytic subunit, catalyzed by upstream kinases such as liver kinase B (LKB1)^[^ ^19^ ^]^ and Ca^2+^/calmodulin–activated kinase 2 (CaMKK2).^[^ ^20^ ^]^ *Foxm1* knockout in mice predominantly reduced the protein expression levels of both phosphorylated and total LKB1 without affecting the expression of CAMKK2 (Figure 4B). Moreover, this *Foxm1* overexpression‐induced AMPK signaling activation in NVCMs was abolished by the LKB1 inhibitor, MDK‐2275, but not by the CaMKK2 inhibitor, STO‐609 (Figure 4C; Figure S3C, Supporting Information). These findings indicate that FOXM1 activates the AMPK signaling pathway through LKB1.

### 2.5 FOXM1 Regulates LKB1 Expression in a Proteasome‐Dependent Manner

Next, we aimed to explore the molecular mechanisms connecting FOXM1 and LKB1. However, CM‐specific *Foxm1* knockout had no effect on *Lkb1* mRNA levels (Figure 4D). Chromatin immunoprecipitation (ChIP)‐quantitative PCR (qPCR) assay revealed that there were no FOXM1 binding sites in the promoter region of *Lkb1* (Figure 4E). These findings suggest that FOXM1 may regulate LKB1 expression in post‐transcriptional level. Next, the ubiquitin‐proteasome system proteasome inhibitor, MG132, and the lysosomal pathway inhibitor, chloroquine (CQ), were used to determine possible mechanisms by which FOXM1 may post‐translationally regulate LKB1 expression. MG132 inhibited LKB1 degradation in the presence of cycloheximide (CHX), but CQ failed to do so (Figure 4F). Moreover, *Foxm1* knockdown in NVCMs significantly reduced the half‐life of LKB1 after CHX treatment, whereas *Foxm1* overexpression markedly increased its half‐life (Figure 4G,H). These findings collectively suggest that FOXM1 may regulate LKB1 degradation in a proteasome‐dependent manner.

Ubiquitination is a process that transfers ubiquitin molecules to substrate proteins to precede its proteasomal degradation. To further validate the ubiquitination of LKB1 and examine its ubiquitination levels and specific sites, we conducted a quantitative ubiquitinome analysis in CMs isolated from the *Myh6* ^+^ and *Foxm1* ^fl/fl^ *Myh6* ^+^ mice (Figure 4I). The analysis identified 376 ubiquitinated proteins, 994 ubiquitinated peptides, and 967 ubiquitinated sites (Figure S4A, Supporting Information). We further analyzed the number of proteins corresponding to different molecular weights as well as the distribution of ubiquitination sites corresponding to ubiquitinated proteins and peptide fragments (Figure S4B–D, Supporting Information). Finally, with a criterion of ≥ 1.2‐fold change between the *Myh6* ^+^ and *Foxm1* ^fl/fl^ *Myh6* ^+^ samples, we obtained 192 significantly increased and 34 significantly decreased ubiquitin sites (Figure S4E, Supporting Information). Differentially ubiquitinated site motifs were analyzed using MoMo (http://meme‐suite.org/tools/momo) (Figure S4F, Supporting Information). To identify ubiquitination sites that may lead to protein degradation, we integrated ubiquitinome analysis with prior quantitative proteomic data (Figure 4I). We identified 11 proteins with decreased expression and increased ubiquitination sites, among which LKB1 was ubiquitinated at Lys146 (Figure 4I,J). These findings suggest the key role of FOXM1 in ubiquitination‐linking of Lys146 residue on LKB1.

### 2.6 FOXM1 Regulates AMPK Via MKRN1‐Mediated LKB1 Ubiquitination

**Figure 5:**
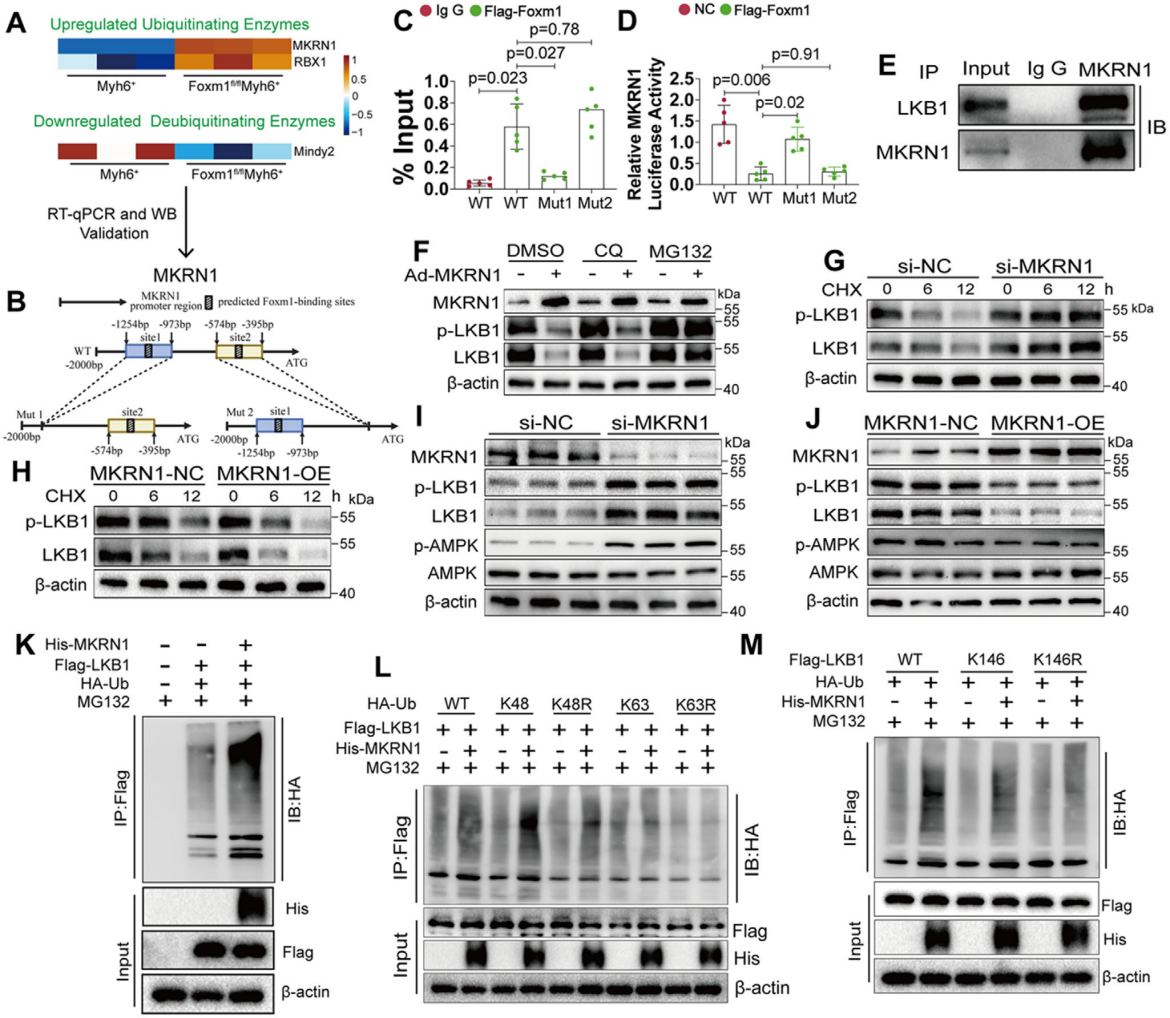
FOXM1 Regulates AMPK via MKRN1‐Mediated LKB1 Ubiquitination. A) Heatmaps of upregulated ubiquitinating enzymes and downregulated deubiquitinating enzymes in cardiomyocytes isolated from *Myh6* ^+^ and *Foxm1^fl/fl^Myh6^+^* mice based on MS data. B) Construction of plasmids containing predicted FOXM1 binding sites within the *Mkrn1* promoter, as identified by the JASPAR database. C) ChIP‐qPCR analysis detected the enrichment of FOXM1 at the Mkrn1 promoter region in AC16 cells co‐transfected with mutated *Mkrn1* promoter plasmids and Flag‐Foxm1 plasmid (*n* = 5). D) Dual luciferase reporter assay in AC16 cells co‐transfected with NC or Flag‐Foxm1 and mutated *Mkrn1* promoter‐driven firefly luciferase reporter plasmids (*n* = 5). E) Endogenous co‐immunoprecipitation of MKRN1 and LKB1 in cardiomyocytes isolated from *Foxm1^fl/fl^Myh6^+^* mice. F) Western blot of MKRN1, p‐LKB1, and LKB1 in NVCMs cultured with DMSO, lysosome inhibitor chloroquine (CQ, 50 µm), and proteasome inhibitor MG132 (50 µm) after infecting with adenovirus expressing MKRN1 or NC (*n* = 3). G) Western blot of p‐LKB1 and LKB1 in NVCMs transfected with si‐NC or si‐*Mkrn1*; and then, treated with CHX for the indicated durations (*n* = 3). H) Western blot of p‐LKB1 and LKB1 in NVCMs infected with adenovirus expressing MKRN1 or NC; and then, treated with cycloheximide (CHX, 50 µm) for the indicated durations (*n* = 3). I) Western blot of MKRN1, p‐LKB1, LKB1, p‐AMPK, and AMPK in NVCMs transfected with si‐NC or si‐*Mkrn1* (*n* = 3). J) Western blot of MKRN1, p‐LKB1, LKB1, p‐AMPK, and AMPK in NVCMs infected with adenovirus expressing MKRN1 or NC (*n* = 3). K) HEK293T cells were transfected with His‐MKRN1, Flag‐LKB1, and HA‐ubiquitin and treated with MG132 (50 µm). Cellular extracts from HEK293T cells were pulled down with anti‐Flag antibody; and then, analyzed by Western blot with anti‐HA antibody. L) HEK293T cells were transfected with His‐MKRN1, Flag‐LKB1, and HA‐tagged mutant ubiquitin, and treated with MG132 (50 µm). Cellular extracts from HEK293T cells were pulled down with anti‐Flag antibody; and then, analyzed by Western blot with anti‐HA antibody (*n* = 3). M) HEK293T cells were transfected with His‐MKRN1, Flag tagged mutated LKB1, and HA‐ubiquitin, and treated with MG132 (50 µm). Cellular extracts from HEK293T cells were pulled down with anti‐Flag antibody; and then, analyzed by Western blot with anti‐HA antibody (*n* = 3). For all statistical plots, the data are presented as mean ± SD. (C,D) By one‐way ANOVA with Bonferroni multiple comparison test.

The close correlation between the ubiquitination and stability of LKB1 prompted us to identify the proteins involved in the regulation of ubiquitination. To further explore the underlying molecular mechanism of FOXM1 deficiency‐induced LKB1 ubiquitination, we evaluated the upregulated ubiquitinating enzymes (UBEs) and downregulated deubiquitinating enzymes (DUBs) from our MS data comparing CMs from *Foxm1^fl/fl^Myh6^+^* and *Myh6^+^* mice. Two critical UBEs, including MKRN1 and ring box protein 1 (RBX1), were significantly upregulated following FOXM1 deficiency; while, only one DUB, Ubiquitin carboxyl‐terminal hydrolase MINDY‐2 (Mindy2), was downregulated (**Figure** **5**A). Next, qRT‐PCR and Western blot analyses were conducted to validate the bioinformatics findings. qRT‐PCR results indicated that conditional knockout of *Foxm1* in CMs increased the mRNA levels of *Mkrn1* and *Rbx1*, whereas *Mindy2* remained unaffected (Figure S5A, Supporting Information). However, Western blot revealed that only the protein levels of MKRN1 were increased in isolated CMs from the *Foxm1* ^fl/fl^ *Myh6* ^+^ mice (Figure S5B, Supporting Information). Next, we investigated the specific promoter motifs of *MRKN1* to which FOXM1 binds. The JASPAR database identified two predicted FOXM1 binding sites within the *MRKN1* promoter which were located from −1045 to −1051 and from −539 to −545, respectively. To experimentally validate these findings, WT and mutant (MUT1 and MUT2) versions of the *MRKN1* promoter were cloned into plasmids and transfected into AC16 cells following FOXM1 overexpression (Figure 5B). Mutation of the −1045 to −1051 site significantly reduced FOXM1 binding efficiency to the *MRKN1* promoter (Figure 5C). Consistently, luciferase reporter assays demonstrated that FOXM1 failed to suppress the activity of the MUT1‐*MRKN1* promoter (Figure 5D). These results indicate the promoter region between −1045 and −1051 base pair is a critical site for FOXM1‐mediated regulation of MKRN1 expression.

Our results prompted us to investigate the involvement of MKRN1 in the ubiquitylation process of LKB1. Co‐immunoprecipitation assay further confirmed a physical interaction between endogenous MKRN1 and LKB1 in *Foxm1* ^fl/fl^ *Myh6* ^+^ CMs (Figure 5D). Indeed, the MKRN1‐induced LKB1 degradation was completely blocked by MG132, but not CQ (Figure 5E). Further, *Mkrn1* knockdown markedly increased the half‐life of LKB1 after CHX treatment (Figure 5F), whereas *Mkrn1* overexpression significantly reduced its half‐life (Figure 5G). Moreover, *Mkrn1* knockdown increased the protein expression of phosphorylated LKB1, AMPK, as well as total LKB1 (Figure 5H), whereas *Mkrn1* overexpression resulted in the opposite effect (Figure 5I). In HEK293T cells, immunoprecipitation and subsequent Western blot analysis demonstrated elevated LKB1 ubiquitination after MKRN1 overexpression (Figure 5J). Altogether, these data suggest that MKRN1 regulates the AMPK signaling pathway by mediating the ubiquitination of LKB1.

To investigate the type of polyubiquitination mediated by MKRN1, cells were transfected with plasmids expressing HA‐tagged mutant ubiquitin (HA‐ubiquitin [K48] or [K63]). The ubiquitin was mutated by substituting all the lysine residues with arginine, except K48 or K63. MKRN1 was able to catalyze LKB1 polyubiquitination with HA‐tagged wild‐type ubiquitin (HA‐ubiquitin (WT)) and HA‐ubiquitin (K48); however, this did not occur with HA‐ubiquitin (K63) or HA‐ubiquitin (K48R) (Figure 5K). The previous quantitative ubiquitinome analysis revealed that LKB1 was ubiquitinated at Lys146 (Figure 4J). To identify the specific lysine residue (Lys146) responsible for the MKRN1‐mediated polyubiquitination of LKB1, we completely mutated LKB1 by substituting all of its lysine residues with arginine (LKB1‐K0). Then, we reintroduced individual lysine residues into LKB1‐K0 to generate LKB1 variants with single‐lysine mutations. Co‐transfection and co‐immunoprecipitation assays in HEK293T cells demonstrated that MKRN1 was able to polyubiquitinate wild‐type LKB1 (LKB1 [WT]) and the LKB1 mutants with conserved Lys146 sites (LKB1 (K146)); however, this did not occur with the LKB1 mutant with mutated Lys146 sites (LKB1 [K146R]) (Figure 5L). Altogether these data demonstrate that MKRN1 catalyzes the K48‐linked polyubiquitination of LKB1 at Lys146.

### 2.7 *Mkrn1* Knockout Ameliorates FOXM1 Deficiency‐Induced Cardiac Dysfunction by Preserving Mitochondrial Bioenergetics

**Figure 6:**
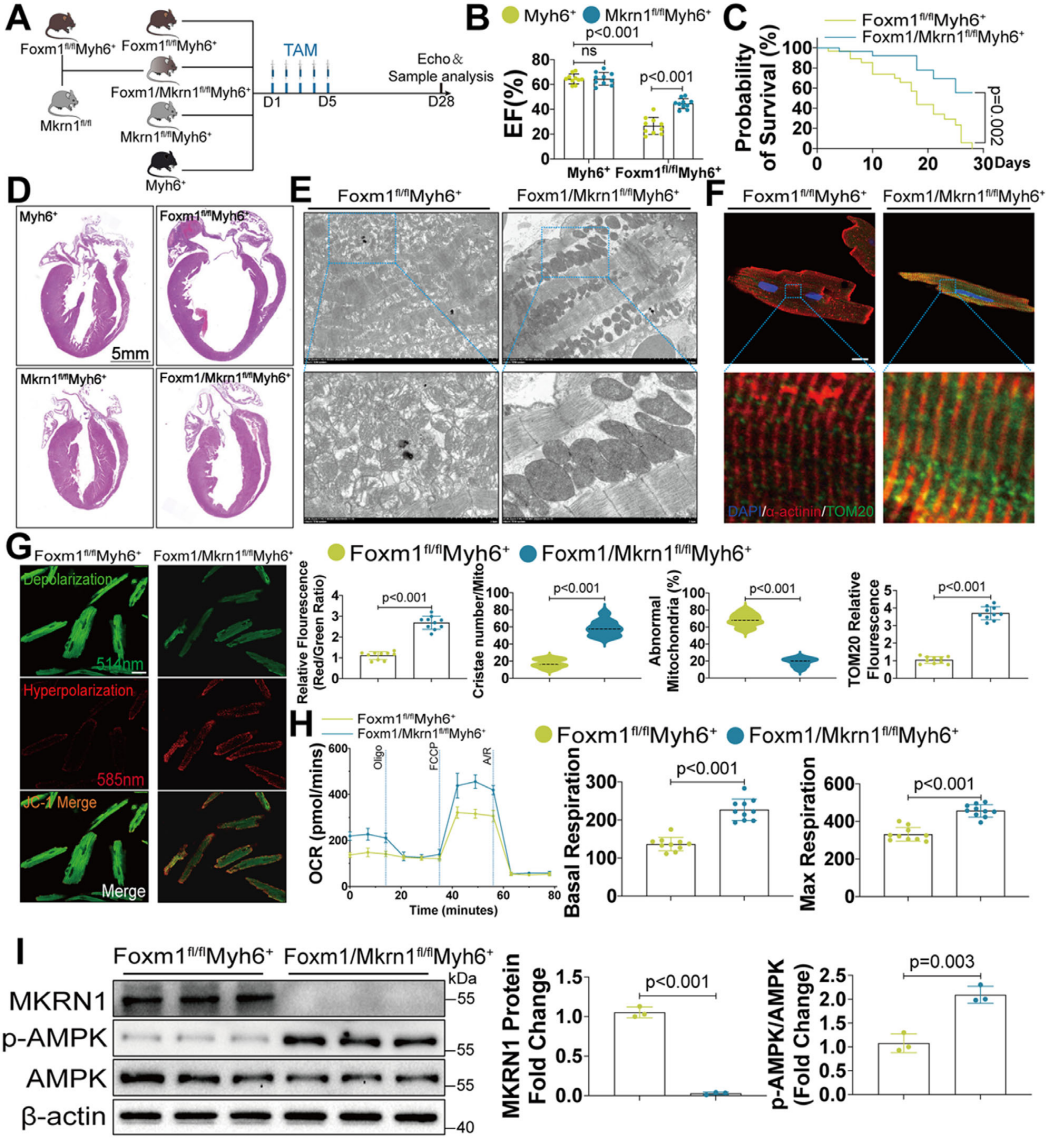
Deleting Mkrn1 attenuates Foxm1 deficiency‐induced heart failure A) Schematic diagram showing the experimental design for the construction of *Foxm1/ Mkrn1^fl/fl^Myh6^+^* mice. B) Ejection fraction (EF) of *Myh6^+^*, *Mkrn1^fl/fl^Myh6^+^*, *Foxm1^fl/fl^Myh6^+^*, and *Foxm1/Mkrn1^fl/fl^Myh6^+^* mice (*n* = 10). C) Survival rate was estimated by Kaplan–Meier method and compared by log‐rank test. D) Longitudinal morphology of hearts from *Myh6^+^*, *Mkrn1^fl/fl^Myh6^+^*, *Foxm1^fl/fl^Myh6^+^*, and *Foxm1/Mkrn1^fl/fl^Myh6^+^* mice by HE staining (*n* = 10, scale bar = 5 mm). E) Representative electron microscopy images and quantitative analysis of mitochondrial from cardiac tissues in *Foxm1^fl/fl^Myh6^+^* and *Foxm1/Mkrn1^fl/fl^Myh6^+^* mice (*n* = 10; scale bar = 5 µm in upper panel and 2 µm in lower panel). F) Immunofluorescence revealed mitochondrial morphology in cardiomyocytes isolated from *Foxm1^fl/fl^Myh6^+^* and *Foxm1/Mkrn1^fl/fl^Myh6^+^* mice (*n* = 10; scale bar = 10 µm in upper panel and 1 µm in lower panel). G) Representative confocal images of mitochondrial transmembrane potential (Δ*ψm*) labeled by JC‐1 monomer (green, marking depolarization) and JC‐1 aggregate (red, marking hyperpolarization) in cardiomyocytes isolated from *Foxm1^fl/fl^Myh6^+^* and *Foxm1/Mkrn1^fl/fl^Myh6^+^* mice (*n* = 10; scale bar = 20 µm). H) Seahorse real‐time traces and averaged data for mitochondrial oxygen consumption rate (OCR) were measured in cardiomyocytes isolated from *Foxm1^fl/fl^Myh6^+^* and *Foxm1/Mkrn1^fl/fl^Myh6^+^* mice using Seahorse XFe24 Analyzer (*n* = 10). I) Western blot and quantification of MKRN1, p‐AMPK, and AMPK protein levels in cardiomyocytes isolated from *Foxm1^fl/fl^Myh6^+^* and *Foxm1/Mkrn1^fl/fl^Myh6^+^* mice (*n* = 3). For all statistical plots, the data are presented as mean ± SD. (B) By two‐way ANOVA with Bonferroni multiple comparison test. (C) By log‐rank test. (E–G) By Welch's *t*‐test. (H,I) By two‐tailed unpaired Student's *t*‐test.

As our in vitro results demonstrated that LKB1 ubiquitination was inhibited by MKRN1 knockdown, we subsequently investigated the contribution of the FOXM1‐MKRN1‐LKB1 axis to cardiac function in vivo. CM‐specific *Mkrn1* knockout mice were generated and crossed with the *Foxm1^fl/fl^Myh6* ^+^ mice to produce the CM‐specific *Foxm1* and *Mkrn1* double‐knockout (DKO; *Foxm1^fl/fl^Mkrn1^fl/fl^Myh6* ^+^) mice (Figure S6A, Supporting Information; **Figure** **6**A). PCR analysis was used to determine the genotypes of *Mkrn1*‐knockout (*Mkrn1^fl/fl^*) mice (Figure S6B, Supporting Information). Reduced FOXM1 and MKRN1 expression was observed after 5 days of TAM administration in the *Foxm1^fl/fl^Mkrn1^fl/fl^Myh6* ^+^ mice compared with the control *Myh6^+^* mice (Figure S6C,D, Supporting Information), and this decrease was specific in CMs (Figure S6E,F, Supporting Information). *Mkrn1* knockout ameliorated the cardiac remodeling and dysfunction observed in the *Foxm1^fl/fl^Myh6* ^+^ mice, as indicated by an increased overall EF (Figure 6B) and IVS (Figure S7A, Supporting Information) as well as a lower overall HW/BW (Figure S7B, Supporting Information). Detailed cardiac ultrasound data are provided in Table S6, Supporting Information. The *Foxm1^fl/fl^Mkrn1^fl/fl^Myh6* ^+^ mice also exhibited an improved survival rate compared with the *Foxm1^fl/fl^Myh6* ^+^ mice (Figure 6C). Further, these mice exhibited a lesser extent of maladaptive hypertrophy and cardiac fibrosis, which was demonstrated by hematoxylin and eosin (H&E), WGA, and picrosirius red staining (Figure 6D; Figure S7C,D, Supporting Information). At the molecular level, *Mkrn1* knockout reduced the expression of the fibrotic markers, Fibronectin and Collagen I, and molecular biomarkers of cardiac hypertrophy, ANP and BNP (Figure S7E, Supporting Information). Next, we examined the effects of MKRN1 on mitochondrial morphology and function. Electron microscopy of the myocardial tissues of the *Foxm1^fl/fl^Mkrn1^fl/fl^Myh6* ^+^ mice revealed markedly improved mitochondrial structure, including reduced number of abnormal mitochondria and increased number of mitochondrial cristae (Figure 6E). Moreover, immunofluorescence analysis of isolated CMs demonstrated that *Mkrn1* knockout significantly increased mitochondrial quantity (Figure 6F); while, reducing mitochondrial depolarization (Figure 6G) and ROS levels (Figure S7F, Supporting Information). In addition, assessment of mitochondrial bioenergetics revealed that *Mkrn1* knockout increased oxygen consumption rate (OCR) of the mitochondrial ETC complex (Figure 6H). At the molecular level, Western blot confirmed that *Mkrn1* knockout upregulated phosphorylated AMPK as well as the ETC complex proteins in isolated CMs, indicating that MKRN1 was responsible for the inactivated AMPK signaling in *Foxm1^fl/fl^Myh6* ^+^ CMs (Figure 6I; Figure S7G, Supporting Information). Altogether, these data confirm that FOXM1 contributes to the balance of mitochondrial bioenergetics via a MKRN1‐dependent pathway and that loss of MKRN1 ameliorates FOXM1 deficiency‐induced cardiac dysfunction.

### 2.8 CM‐Specific *Foxm1* Overexpression Protects Against Myocardial I/R Injury in Mice

**Figure 7:**
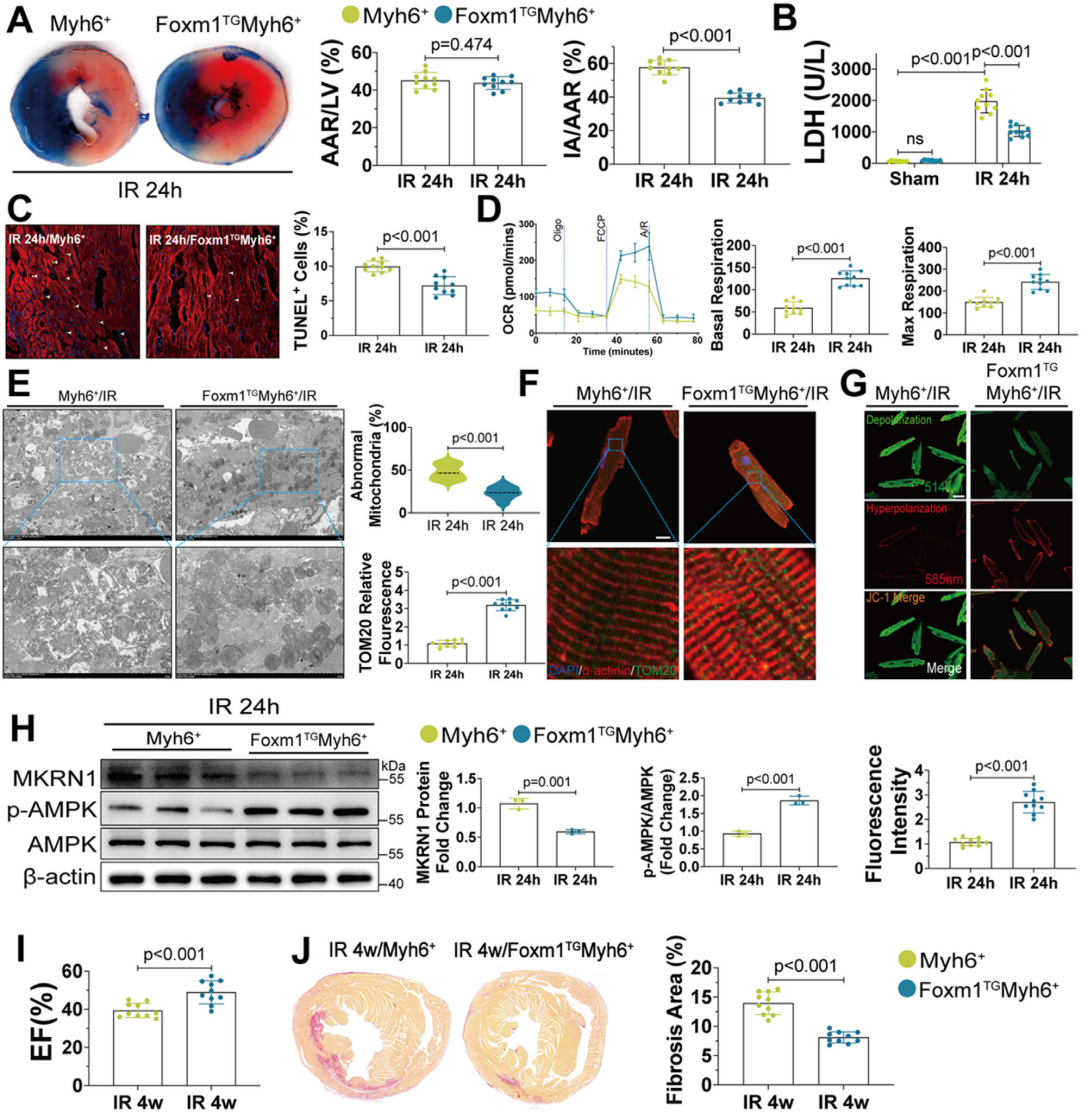
Foxm1 overexpression ameliorates I/R‐induced cardiac injury in mice. A) Representative images of heart sections stained by Evans blue and TTC at 24 h after myocardial‐ischemia reperfusion injury from *Myh6* ^+^ and *Foxm1* ^TG^ *Myh6* ^+^ mice. The ratio of area at risk (AAR) to left ventricle (LV) and infarct area (IA) to AAR were calculated based on Evans blue and TTC staining (*n* = 10). B) The serum levels of lactate dehydrogenase (LDH, U/L) of *Myh6* ^+^ and *Foxm1* ^TG^ *Myh6* ^+^ mice 24 h after I/R surgery (*n* = 10). C) Representative TUNEL staining and relative quantification in heart samples of *Myh6* ^+^ and *Foxm1* ^TG^ *Myh6* ^+^ mice 24 h after I/R surgery (*n* = 10; scale bar = 20 µm). D) Seahorse real‐time traces and averaged data for mitochondrial oxygen consumption rate (OCR) were measured in cardiomyocytes isolated from *Myh6* ^+^ and *Foxm1* ^TG^ *Myh6* ^+^ mice 24 h after I/R surgery using Seahorse XFe24 Analyzer (*n* = 10). E) Representative electron microscopy images and quantitative analysis of mitochondria from cardiac tissues in *Myh6* ^+^ and *Foxm1* ^TG^ *Myh6* ^+^ mice 24 h after I/R surgery (*n* = 10; scale bar = 5 µm in upper panel and 2 µm in lower panel). F) Immunofluorescence revealed mitochondrial morphology in cardiomyocytes isolated from *Myh6* ^+^ and *Foxm1* ^TG^ *Myh6* ^+^ mice 24 h after I/R surgery (*n* = 10; scale bar = 10 µm in upper panel and 1 µm in lower panel). G) Representative confocal images of mitochondrial transmembrane potential (Δ*ψm*) labeled by JC‐1 monomer (green, marking depolarization) and JC‐1 aggregate (red, marking hyperpolarization) in cardiomyocytes isolated from *Myh6* ^+^ and *Foxm1* ^TG^ *Myh6* ^+^ mice 24 h after I/R surgery (*n* = 10; scale bar = 20 µm). H) Western blot and quantification of MKRN1, p‐AMPK, and AMPK protein levels in cardiomyocytes isolated from *Myh6* ^+^ and *Foxm1* ^TG^ *Myh6* ^+^ mice 24 h after I/R surgery (*n* = 3). I) Ejection fraction (EF) of *Myh6* ^+^ and *Foxm1* ^TG^ *Myh6* ^+^ mice 4 weeks after I/R surgery (*n* = 10). J) Picrosirius red staining of heart sections of *Myh6* ^+^ and *Foxm1* ^TG^ *Myh6* ^+^ mice 4 weeks after I/R surgery (*n* = 10; scale bar = 5 mm). For all statistical plots, the data are presented as mean ± SD. (A,C,D,H–J) By two‐tailed unpaired Student's *t*‐test. (B) By Welch's ANOVA with Dunnett's T3 multiple comparisons test. (E–G) By Welch's *t*‐test.

Considering the critical role of FOXM1 in energy metabolism and cardiac function, we next investigated whether *Foxm1* overexpression could ameliorate myocardial I/R injury by improving cardiac energy metabolism. *Foxm1*‐transgenic (*Foxm1* ^TG^) mice were crossed with *Myh6*‐Cre mice to generate CM‐specific *Foxm1* overexpression mice (*Foxm1* ^TG^ *Myh6* ^+^, Figure S8A, Supporting Information). PCR analysis was used to determine the genotypes of the mice (Figure S8B, Supporting Information). FOXM1 mRNA and protein levels were evaluated in the *Foxm1* ^TG^ *Myh6* ^+^ mice to confirm successful overexpression (Figure S8C,D, Supporting Information). CM‐specific overexpression was confirmed by qRT‐PCR and western blot (Figure S8E‐G, Supporting Information). HE and WGA staining demonstrated FOXM1 overexpression did not alter heart size or cardiomyocyte number under basal conditions (Figure S8H,I, Supporting Information). Further, FOXM1 overexpression had no significant effect on the expression levels of cell cycle‐associated markers (Figure S8J, Supporting Information). These mice were then subjected to 40 min of myocardial ischemia, followed by either 24 h or 28 days of reperfusion to assess short‐term and long‐term effects, respectively. The overall area at risk (AAR)/left ventricle (LV) was comparable between the *Myh6* ^+^ and *Foxm1* ^TG^ *Myh6* ^+^ mice, whereas the *Foxm1* ^TG^ *Myh6* ^+^ mice exhibited a much smaller overall infarct area (IA)/AAR than the *Myh6* ^+^ mice after 24 h of I/R (**Figure** **7**A). In addition, I/R‐induced lactate dehydrogenase (LDH) release was also significantly reduced in the *Foxm1* ^TG^ *Myh6* ^+^ mice (Figure 7B). TUNEL staining revealed that Foxm1 overexpression significantly reduced I/R induced CM apoptosis (Figure 7C). To further investigate whether *Foxm1* overexpression would enhance mitochondrial energy metabolism following I/R, we isolated CMs and collected myocardial tissues from ischemic region of *Foxm1* ^TG^ *Myh6* ^+^ and *Myh6* ^+^ mice after 24 h I/R. Seahorse analysis showed that *Foxm1* overexpression improved oxidative respiration in isolated CMs (Figure 7D). Electron microscopy of the myocardial tissues from the *Foxm1* ^TG^ *Myh6* ^+^ mice revealed markedly improved mitochondrial structure, including reduced abnormal mitochondria number and increased mitochondrial cristae number (Figure 7E). Immunofluorescence and Western blot analyses demonstrated that *Foxm1* overexpression significantly increased mitochondrial quantity and upregulated the expression of ETC complex proteins (Figure 7F; Figure S9A, Supporting Information). As a result, the *Foxm1* ^TG^ *Myh6* ^+^ CMs exhibited decreased mitochondrial depolarization and ROS level (Figure 7G; Figure S9B, Supporting Information). Moreover, Western blot confirmed that *Foxm1* overexpression upregulated phosphorylated AMPK in isolated CMs (Figure 7H), indicating that the beneficial effects mediated by FOXM1 were at least partially through the MKRN1‐LKB1‐AMPK axis.

Next, we assessed cardiac function and adverse cardiac remodeling in the *Myh6* ^+^ and *Foxm1* ^TG^ *Myh6* ^+^ mice at 28 days after I/R injury. Sirius red staining showed that the *Foxm1* ^TG^ *Myh6* ^+^ mice exhibited significantly reduced I/R‐induced cardiac fibrosis (Figure 7J). Consistently, the *Foxm1* ^TG^ *Myh6* ^+^ mice exhibited significantly preserved EF and FS values after I/R injury compared with those of the *Myh6* ^+^ mice (Figure 7I; Figure S9C, Supporting Information). A significant reduction of fibrotic markers, Fibronectin and Collagen I, and molecular biomarkers of cardiac hypertrophy, ANP and BNP, was also observed in the *Foxm1* ^TG^ *Myh6* ^+^ mice after long term I/R injury (Figure S9D, Supporting Information). Detailed cardiac ultrasound data are provided in Table S7, Supporting Information. These results suggest that *Foxm1* overexpression in CMs may be an effective strategy to protect against myocardial I/R injury and mitochondrial dysfunction.

### 2.9 *Foxm1* Overexpression Protects Against Myocardial I/R Injury in Bama Pigs

**Figure 8:**
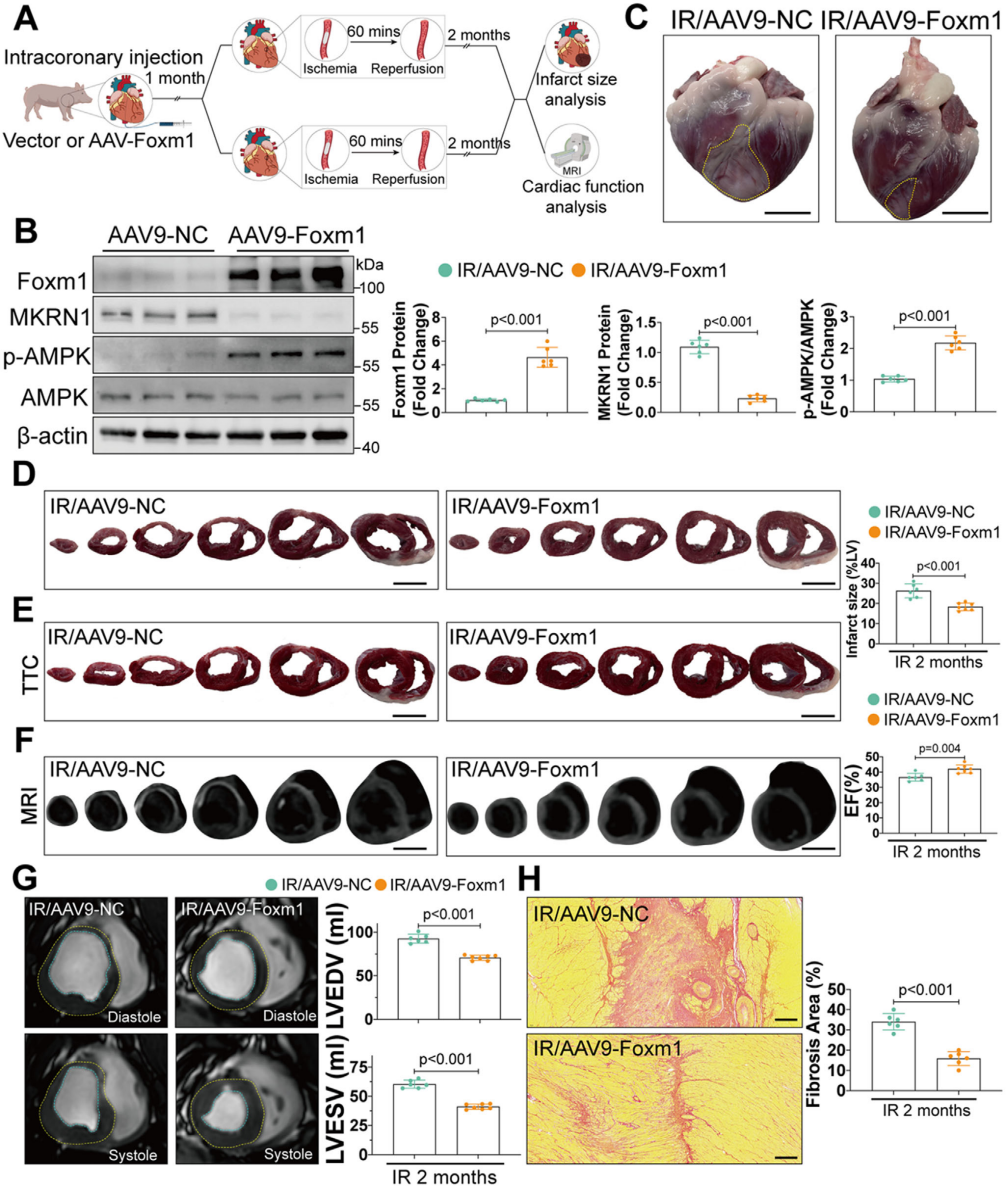
Overexpressing FOXM1 mitigates I/R‐induced heart failure in pigs. A) Schematic diagram illustrating experimental design of pigs. After intracoronary injection of FOXM1‐overexpressing AAV (AAV9‐Foxm1) or control AAV (AAV9‐NC), pigs were subjected to I/R injury (ischemia 60 min before reperfusion). The alive pigs were observed for 2 months, after which MRI examination and histological analysis for cardiac function and morphology were conducted. B) Western blot and quantification of FOXM1, MKRN1, p‐AMPK, and AMPK in cardiac tissues of pigs subjected to AAV9‐Foxm1 or AAV9‐NC administration (*n* = 3). C) Representative morphology of intact hearts of pigs at 2 months after I/R surgery with AAV9‐Foxm1 or AAV9‐NC administration (*n* = 6; scale bar = 2.5 cm). D) Representative heart sections of pigs at 2 months after I/R surgery with AAV9‐Foxm1 or AAV9‐NC administration (*n* = 6; scale bar = 3 cm). E) Representative TTC staining of heart sections of pigs at 2 months after I/R surgery with AAV9‐Foxm1 or AAV9‐NC administration (*n* = 6; scale bar = 3 cm). F) Representative heart images of magnetic resonance imaging coronal views of pigs at 2 months after I/R surgery with AAV9‐Foxm1 or AAV9‐NC administration (*n* = 6; scale bar = 3 cm). G) Representative heart images of magnetic resonance imaging transversal views in systolic and diastolic phases of pigs at 2 months after I/R surgery with AAV9‐Foxm1 or AAV9‐NC administration (*n* = 6; scale bar = 3 cm). LVEDV, left ventricular end‐diastolic volume. LVESV, left ventricular end‐systolic volume. H) Picrosirius red staining and quantification from heart samples of pigs at 2 months after I/R surgery with AAV9‐Foxm1 or AAV9‐NC administration (*n* = 6; scale bar = 100 µm). For all statistical plots, the data are presented as mean ± SD. (B,E–H) By two‐tailed unpaired Student's *t*‐test.

Although mice have been widely used to study the pathological features and molecular mechanisms of various diseases, the clinical translation is often limited due to the physiological and structural differences between rodents and humans.^[^ ^21^ ^]^ Therefore, large animal models, such as pigs, often serve as a better model in terms of translational study.^[^ ^22^ ^]^ Therefore, to further investigate the translational potential of targeting FOXM1, we established an I/R injury model in Bama pigs by conducting LAD coronary artery occlusion for 60 min followed by reperfusion for 2 months (Figure S10A, Supporting Information). Pigs were injected with AAV9‐FOXM1 or AAV9‐control (NC) virus via the coronary artery at 1 month before I/R surgery. Tissue and cardiac function analyses were performed at 2 months after I/R surgery (**Figure** **8**A). Results showed that the use of AAV9 did not affect the physiological function of major organs in pigs through biochemical analyses. We extracted lysates from different organs to examine the distribution of FOXM1 protein after AAV9‐FOXM1 injection and found that it was predominantly expressed in the heart, besides a slight increase in the liver (Figure S10B, Supporting Information). High FOXM1 expression levels were detected in different cardiac segments dominated by the LAD (left anterior descending), LCX (left circumflex), and RCA (right coronary artery) after AAV9‐FOXM1 injection (Figure S10B, Supporting Information). Moreover, Western blot confirmed that FOXM1 overexpression also led to downregulation of MKRN1 and activation of the AMPK signaling pathway in the myocardial tissues of these pigs (Figure 8B). This analysis revealed the antegrade delivery of AAV9 to the heart via the coronary artery as an effective delivery method in pigs.

Gross view of heart and triphenyl tetrazolium chloride (TTC) staining demonstrated that the pigs injected with AAV9‐FOXM1 displayed reduced infarct sizes after I/R surgery (Figure 8C–E). Reduced infarct sizes were also evidenced by late‐enhancement magnetic resonance imaging (MRI) (Figure 8F). Likewise, MRI showed that AAV9‐FOXM1 injection significantly improved systolic and diastolic cardiac function in pigs after I/R surgery, compared with controls (Figure 8G). In addition, Sirius red staining showed that these pigs exhibited significantly reduced cardiac fibrosis (Figure 8H). A significant reduction in fibrotic markers Fibronectin and Collagen I and cardiac hypertrophy biomarkers ANP and BNP was also observed in the pigs with FOXM1 overexpression after I/R surgery (Figure S10C, Supporting Information). Moreover, sustained upregulation of FOXM1 expression was observed in cardiac tissue 6 months following AAV administration (Figure S10D, Supporting Information). Assessments of liver, kidney, and cardiac safety markers further revealed no indications of significant toxicity (Figure S10E, Supporting Information). These findings suggest that FOXM1 overexpression may serve as an effective preventive strategy against myocardial I/R injury in large animal models.

## 3 Discussion

Mitigating cardiac injury induced by revascularization remains an overlooked therapeutic objective but a pressing clinical necessity.^[^ ^23^ ^]^ Despite numerous promising outcomes in experimental studies, the translation of effective treatments for I/R injury into clinical practice has been hindered by the limited clinical relevance of employed models or methodologies. Here, by conducting a comprehensive analysis of cardiac samples obtained from clinical ICM patients and mice subjected to I/R injury, we uncovered a significant correlation between the expression of Foxm1 and the pathogenesis of myocardial ischemia. Further, Foxm1 depletion in postmitotic cardiomyocytes of adult mice was associated with the occurrence of HF. Proteome and ubiquitinome data revealed that *Foxm1* knockout in CMs resulted in enhanced LKB1 ubiquitination and impaired AMPK signaling and energy metabolism. Proteomic analysis identified the E3 ligase, MKRN1, as a key regulator of LKB1 and the AMPK signaling pathway, functioning via the direct conjugation of K48‐linked ubiquitin chains to LKB1 at Lys146. Remarkably, restored FOXM1 expression improved mitochondrial energy metabolism and protected against post‐MI HF in both murine and porcine models. Collectively, these findings demonstrate that FOXM1 is a novel regulator of mitochondrial homeostasis and cardiac function; targeting FOXM1/MKRN1/LKB1/AMPK axis might be a potential therapeutic option to protect against I/R injury and HF.

FOXM1 is a member of the Fox family of TFs, which shares homology in the winged helix/forkhead DNA‐binding domain.^[^ ^24^, ^25^ ^]^ It acts as a key regulator of cell cycle progression in tumor cells during the development of liver, colon, and breast cancer.^[^ ^26^, ^27^, ^28^ ^]^ FOXM1 is expressed in all tissues during embryogenesis, but only low expression levels are required in adult mice. The cell‐specific loss of FOXM1 in different tissues has demonstrated that FOXM1 is essential for mouse embryonic development.^[^ ^15^, ^16^, ^29^, ^30^ ^]^ SMC‐specific FOXM1 loss during the embryonic period results in severe pulmonary hemorrhage and structural defects in arterial walls.^[^ ^17^ ^]^ Global and cardiac‐specific *Foxm1* knockout both cause reduced CM proliferation and embryonic lethality during late gestation.^[^ ^15^, ^16^, ^30^ ^]^ These studies have shown that FOXM1 has distinct roles in different tissues during embryonic development. In response to organ injury, FOXM1 expression is induced in a variety of cell types, including fibroblasts, endothelial cells (ECs), and SMCs, to promote the repair processes. Penke et al. have reported that FOXM1 inhibition attenuates bleomycin‐induced pulmonary fibrosis by reducing the activation of lung fibroblasts.^[^ ^14^ ^]^ In addition, Zhao et al. have demonstrated that EC‐ and SMC‐specific *Foxm1* knockout ameliorate LPS‐induced pulmonary vascular injury and hypoxia‐induced pulmonary hypertension, respectively.^[^ ^31^, ^32^ ^]^ However, the mechanisms through which FOXM1 mediates the progression of HF remain unexplored. A previous report has shown that FOXM1 is not essential for postnatal cardiac development or CM proliferation after postnatal day 7, but it is required for the maintenance of cardiac structure during aging.^[^ ^33^ ^]^ Here, we have revealed that cardiac FOXM1 protein levels are clearly upregulated in cardiac samples of ICM patients as well as in a mouse model of I/R injury. By engineering CM‐specific *Foxm1*‐knockout and ‐transgenic mice, we have demonstrated that CM‐derived FOXM1 protects cardiac function by preserving mitochondrial bioenergetics. The discrepancy between the two studies may be due to the different knockout strategies. In the Bolte study,^[^ ^33^ ^]^ LoxP sequences flanked exon 4–7 of the *Foxm1* gene, whereas knockout of exon 3 resulted in a frameshift of the *Foxm1* gene in our study. Further, different Cre‐LoxP technologies were used for conditional gene loss (αMHC‐Cre in the Bolte study and inducible αMHC‐MerCreMer in our study).^[^ ^34^ ^]^ Therefore, more in‐depth research using chromatin‐surveying techniques to determine the function and mechanisms of FOXM1 in the cardiovascular system is warranted.

During HF, the heart faces an energy deficit, which is primarily due to decreased mitochondrial oxidative capacity.^[^ ^35^ ^]^ In colon cancer, it has been shown that FOXM1 inhibition has the potential to result in mitochondrial dysfunction and enhance sensitization to anticancer drugs.^[^ ^28^ ^]^ Researchers have also found evidence supporting a critical role of FOXM1 in the regulation of aerobic glycolysis.^[^ ^36^, ^37^ ^]^ In addition, Black et al. have suggested that FOXM1 is translocated into mitochondria, which is a critical process independent of nuclear transcription for mitochondrial homeostasis.^[^ ^18^ ^]^ These studies suggest an important role of FOXM1 in the regulation of the mitochondrial genome and function. The heart has a very high energy demand and must continuously produce large amounts of ATP to sustain contractile function. The heart achieves this primarily via mitochondrial oxidative phosphorylation and by metabolizing a variety of fuels, including fatty acids, glucose, lactate, ketones, pyruvate, and amino acids. However, to date, the role and underlying mechanisms of FOXM1 in HF, particularly pertaining to mitochondrial energetics, remain poorly understood. Here, we have demonstrated that CM‐specific *Foxm1* knockout leads to the dysregulation of mitochondrial biogenesis, resulting in significant impairment of contractile function and HF. To the best of our knowledge, this is the first study to report FOXM1 as a metabolic regulator during HF progression. FOXM1 expression has also been associated with pathological conditions in different cell types. Therefore, defining the mechanistic details of FOXM1‐mediated regulation, as well as of its upstream regulators, will help to better understand the molecular mechanisms underlying certain diseases and to develop therapeutic interventions.

AMPK acts as an energy sensor to regulate multiple physiological processes in the cardiovascular system; thus, making it a potential therapeutic target. A substantial body of evidence indicates that upstream kinases, including LKB1, play critical roles in AMPK activation and HF progression via many processes, including the regulation of autophagy, inflammation, and lipid metabolism.^[^ ^38^ ^]^ Consistently, we have observed downregulated LKB1 activity in our conditional *Foxm1*‐knockout mice. However, the activity of calcium‐calmodulin‐dependent kinase kinase 2 (CaMKK2), another upstream kinase of AMPK, remains unaltered in these mice. Research has found that LKB1 can be phosphorylated, deacetylated, and ubiquitinated under different conditions.^[^ ^39^, ^40^, ^41^ ^]^ Several kinases, such as protein kinase A (PKA), protein kinase C (PKC), and ataxia‐telangiectasia mutated (ATM), have been reported to phosphorylate LKB1.^[^ ^39^, ^42^ ^]^ SIRT2 has been shown to bind and deacetylate LKB1 to attenuate angiotensin II‐induced cardiac hypertrophy.^[^ ^40^ ^]^ LKB1 can be post‐translationally modified by ubiquitination and deubiquitination, but not by phosphorylation or prenylation. Sirtuin 1 (SIRT1) has been shown to inhibit AMPK activation by promoting the ubiquitination and proteasomal degradation of LKB1.^[^ ^41^ ^]^ Tankyrases interact with LKB1 to promote its K63‐linked ubiquitination by the E3 ligase, ring finger protein 146 (RNF146), which in turn blocks its activation.^[^ ^43^ ^]^ Recent studies on E3 ligase MKRN1‐mediated ubiquitination and proteasomal degradation of AMPKα have implicated that post‐translational modification of the AMPK signaling pathway regulates protein homeostasis; and thus, imposes significant systemic metabolic effects.^[^ ^44^ ^]^ We have identified MKRN1 as a key regulator of LKB1; and therefore, of the AMPK signaling pathway, that acts through the direct conjugation of K48‐linked ubiquitin chains to LKB1 at Lys146. We have further demonstrated that MKRN1 deficiency in CMs ameliorates cardiac dysfunction and the pathology associated with in vivo FOXM1 deficiency. This study provides a crucial perspective on the importance of the post‐translational regulation of AMPK in metabolic pathways that may help researchers to develop novel therapeutic strategies targeting AMPK or its upstream regulators.

Our data show that acute FOXM1 depletion in CMs leads to systolic HF and mortality, raising the question of whether *Foxm1* overexpression elicits protection against HF in adult mice. Craig et al. have demonstrated that nanoparticle delivery of FOXM1 stimulates lung angiogenesis and alveolarization during recovery from neonatal hyperoxic injury.^[^ ^45^ ^]^ Shuo et al. have found that *Foxm1* overexpression not only attenuates acute myocardial infarction injury, but it also prevents ventricle remodeling and infarct expansion.^[^ ^46^ ^]^ Here, we have generated a TAM‐inducible, CM‐specific, *Foxm1*‐transgenic mouse line to demonstrate that CM‐specific *Foxm1* overexpression attenuates I/R injury‐induced cardiac damage and remodeling by preserving mitochondrial bioenergetics.

Murine models are widely used in cardiovascular research due to their advantages in genetic manipulation, cost‐effectiveness, and accessibility, particularly allowing for the creation of transgenic or knockout models to study specific genes and their functions. However, their clinical translation is limited by discrepancies in cardiac characteristics, including cardiac structure, metabolism, and immune response compared to humans and large animals.^[^ ^22^ ^]^ In contrast, porcine models are often regarded as more physiologically relevant for human cardiac studies due to their analogous heart size, coronary anatomy, and comparable electrophysiological properties.^[^ ^21^, ^22^, ^47^ ^]^ Given the evidence of similar patterns of Foxm1 expression in ischemic heart tissues across humans, mice, and pigs, along with analogous energy metabolic patterns between swine and humans,^[^ ^48^ ^]^ we hypothesize that the Foxm1‐related mechanisms of heart failure in pigs may yield findings with greater translational relevance to human conditions. In summary, we investigate genetic mechanisms using murine models; while, extending the translational relevance of our findings through examination in porcine models.

Viral vectors are widely employed in various preclinical animal models to deliver genetic constructs into target tissues.^[^ ^49^ ^]^ Among these vectors, adeno‐associated viral vectors (AAVs) are gaining prominence for their ability to achieve tissue‐specific targeting based on the selected serotype.^[^ ^50^, ^51^ ^]^ The number of clinical trials for AAV‐based therapies has increased drastically, resulting in over 200 registered clinical trials to date.^[^ ^52^ ^]^ Notably, AAV9 is recognized for its extensive biodistribution and effective transduction capacity in cardiac tissue, owing to its capability to traverse the vascular endothelial barrier,^[^ ^53^ ^]^ thereby rendering it suitable for cardiac‐specific applications such as Foxm1 overexpression. In addition, the incorporation of a cardiac‐specific promoter, such as troponin T (TnT), further ensures tissue‐specific expression, thereby minimizing off‐target expression in non‐cardiac tissues and mitigating potential adverse effects.^[^ ^53^ ^]^ Although intravenous administration is commonly utilized for systemic delivery, intracoronary^[^ ^54^ ^]^ delivery method may be considered to enhance efficiency, particularly when targeting localized areas of the heart. Notably, intracoronary infusion via percutaneous catheter‐based approaches has gained appeal due to its limited invasiveness.^[^ ^55^, ^56^ ^]^ Our translational study has demonstrated the feasibility, long‐term therapeutic effectiveness, and favorable safety profile of cardiac AAV9‐*Foxm1* gene therapy in preclinical models of HF. Altogether, our findings support the application of AAV9‐induced FOXM1 expression as a novel therapy for patients with acute myocardial infarction to potentially prevent the onset of chronic HF.

Our study has several limitations. First, although echocardiography in mice is a widely used technique for assessing cardiac structure and function, it has several limitations due to the unique challenges associated with small animal models. Factors such as anesthesia depth, respiratory rate, heart rate variability, and operator dependency all contribute to decreased accuracy and reproducibility of cardiac ultrasound measurements in mice. It is important to recognize these limitations and use complementary methods (such as MRI or invasive cardiac ultrasound) to validate findings in the future. Second, while the use of a specific AAV serotype and promoter effectively limited transgene expression predominantly to the heart, our findings show that AAV‐mediated FOXM1 overexpression also occurred in the liver. As FOXM1 is a recognized oncogene and has been implicated in the development of liver tumors, this observation raises potential concerns regarding hepatic safety. Consequently, long‐term evaluation of liver function is warranted to identify any associated risks prior to advancing FOXM1‐based therapies toward clinical application. Last, only rodent and porcine animals were used in our study; and thus, the implications of our findings for human application remain uncertain. Future research involving non‐human primates and human iPSC‐derived cardiomyocytes would provide valuable insights and improve translational relevance for myocardial I/R studies.

In conclusion, our study delineates that the FOXM1/MKRN1/LKB1/AMPK axis is an essential regulator to maintain mitochondrial homeostasis and cardiac function. Elevated CM‐derived FOXM1 effectively protects against cardiac dysfunction in mice and pigs, and these findings may provide new mechanistic insights and a potential therapeutic target for treating myocardial I/R injury and HF.

## 4 Experimental Section

### Human Samples

Ischemic heart tissue of ICM patients (*n* = 7) was obtained during cardiac transplantation. Healthy heart samples (*n* = 7) were collected from brain‐dead donors with normal circulatory supply, who were not suitable for transplantation due to the technical or non‐cardiac reasons following the guideline of China Transplant Services. The experiments conformed to the principles set out in the WMA Declaration of Helsinki and the ethical review committee of Zhongshan Hospital, Fudan University (B2022‐267R). All participants provided written informed consent before heart tissue collection. Detailed information of human heart samples is provided in Table S2, Supporting Information.

### Animals

Male mice aged 6–8 weeks on the C57BL/6 background were purchased from SLAC Laboratory Animal Co. Ltd. (Shanghai, China). All mice were housed in standard laboratory mouse chow at 22 °C constant temperature with free access to tap water and a 12:12‐h light: dark cycle environment. In animal studies, mice were randomly distributed to each group using a random number generator. The investigators were blind to treatment/genotype group during the experiment and quantification. All animal experiments were approved by the Animal Care and Use Committee at Zhongshan Hospital, Fudan University, China (Approval Nos. 2023–143 for mice and 2023‐021 for pigs) and were performed in accordance with the standards of the ARRIVE guidelines.

### Genetically Engineered Mice

*Foxm1* conditional knockout mouse model was created by Cyagen Biosciences Inc (Guangzhou, Guangdong, China) using CRISPR/Cas‐mediated genome engineering. The *Foxm1* gene was located on mouse chromosome 6. Eight exons were identified and exon 3 was selected as the conditional knockout region (cKO region). Knockout of Exon 3 would result in frameshift of the gene, and consequently, cause the loss of function of the mouse *Foxm1* gene. PCR for genotyping was performed with primers: 5′‐ GACTTTACTGTCTGGGACCTCTC‐3′ (sense) and 5′‐ TACATTCGATTTGTACTGCTGGGAA‐3′ (antisense), which yielded one band with 258 bp products for *Foxm1^fl/fl^*, two bands with 258 and 186 bp products for *Foxm1^fl/+^*, and one band with 186 bp products for WT.

*Foxm1* conditional knockin mouse model at the locus of ROSA26 was created by Cyagen Biosciences Inc (Guangzhou, Guangdong, China) using CRISPR/Cas‐mediated genome engineering. The mouse *ROSA26* gene and *Foxm1* gene were located on mouse chromosome 6. The “CAG‐loxP‐Stop‐loxP‐Kozak‐mouse *Foxm1* CDS without stop codon‐2A‐EGFP‐polyA” cassette was cloned into intron of *ROSA26* to generate targeted conditional knockin offspring. The expression of mouse *Foxm1* CDS was dependent on Cre recombination. PCR for genotyping was performed with primers: F1: 5′‐ GACTTTACTGTCTGGGACCTCTC‐3′ (sense) and R1: 5′‐ TACATTCGATTTGTACTGCTGGGAA‐3′ (antisense), and F2: 5′‐AGATCTGCAAGCTAATTCCTGC‐3′ (sense) and R2: 5′‐GGTTGATAATCTTGATTCCGGCTG‐3′ (antisense), which yielded one band with 310 bp products for *Foxm1^TG/TG^*, two bands with 310 and 607 bp for *Foxm1^TG/+^*, and one band with 607 bp for WT.

The *Mkrn1* conditional knockout mouse model was created by Cyagen Biosciences Inc (Guangzhou, Guangdong, China) using CRISPR/Cas‐mediated genome engineering. The *Mkrn1* gene was located on mouse chromosome 6. Eight exons were identified and exons 3 and 4 were selected as the knockout region (cKO region). The knockout of ≈Exon 3–4 would result in frameshift of the gene, and consequently, cause the loss of function of the mouse *Mkrn1* gene. PCR for genotyping was performed with primers: F: 5′‐CATCCACTTAGTAGTTGGCCTCTG‐3′ (sense) and R: 5′‐GTGTAACAGCCACACAATCAACAT‐3′ (antisense), which yielded one band with 304 bp products for *Mkrn1^fl/fl^*, two bands with 304 and 239 bp for *Mkrn1^fl/+^*, and one band with 239 bp for WT.

*Myh6*‐MerCreMer mice were obtained from Cyagen Biosciences Inc (Guangzhou, Guangdong, China). *Foxm1^fl/fl^, Foxm1^TG/TG^, and Mkrn1^fl/fl^ mice* were bred with *Myh6*‐MerCreMer mice to achieve CM‐specific *Foxm1* knockout, *Foxm1* overexpression, and *Mkrn1* knockout. Cre expression was induced by daily intraperitoneal injections of 25 mg kg^−1^ tamoxifen (T5648, Sigma–Aldrich) for 5 days. The control cohort of *Myh6*‐MerCreMer mice received an equivalent dosage of tamoxifen administration for standardization purposes.

### Mouse Myocardial Ischemia‐Reperfusion Model

Mice were anesthetized by 2% isoflurane with 100% O_2_ ventilation at 2 L min^−1^. After a left thoracic incision, the heart was exposed and a slipknot was made around the left anterior descending coronary artery with a 6‐0 silk suture. Myocardial ischemia was confirmed by the presence of ST elevations on the electrocardiogram. After 40 min of ischemia, reperfusion was started by releasing the slipknot. The sham‐operated mice underwent only left‐side thoracotomy without LAD ligation. Heart tissue from the IR region of the left ventricular myocardium was collected for qRT‐PCR and Western blot analysis.

### Determination of Mouse Myocardial Infarcted Size

The infarct size and area at risk (AAR) were evaluated by 2,3,5‐triphenyltetrazolium chloride/Evans Blue (TTC/EB) staining. After a 24‐h reperfusion, the mice were anesthetized; and then, the chest was opened. The coronary artery was re‐occluded at the site of occlusion and 1% Evans blue dye was injected from aortic to distinguish normal areas and ischemic areas. The heart was then washed in phosphate‐buffered saline (PBS) and frozen at −80 °C for 15 min before being cut into five 1.2‐mm‐thick transverse slices. Then, the slices were incubated in 1% TTC at 37 °C for 15 min to visualize the unstained infarcted region. The infarct area (pale), AAR (red), and total LV area of each section (blue) were determined using Image‐Pro Plus 6.0 (NIH, Bethesda, MD) by a blinded observer.

### Biochemical and Bioenergetic Assays

For the collection of blood samples, mice were anesthetized by intraperitoneal injection of sodium pentobarbital (50 mg kg^−1^) and were exsanguinated via retro‐orbital puncture under general anesthesia. The blood samples were placed at room temperature for 30 min prior to centrifugation at 1000 × *g* for 5 min. Serum was collected and stored at −80 °C for further analysis. The serum and cultured supernatant levels of lactate dehydrogenase (LDH) in the study were detected by the LDH detection kit (C0016, Beyotime, China) according to the manufacturer's instructions.

Oxygen consumption rate (OCR) of isolated primary cardiomyocytes in indicated groups was analyzed as described previously.^[^ ^57^ ^]^ The metabolic OCR profiles were detected by adding oligomycin A (1 µm), 1 µm FCCP, antimycin A (1 µm), and rotenone (1 µm). Oxidative phosphorylation (OxPhos) was calculated as follows: basal respiration (OCRpre‐Olig), maximal respiration (OCRpost‐FCCP), and spare capacity (OCRpost‐rotenone—OCRpre‐Olig).

### Mitochondrial Membrane Potential (Δ*Ψm*) Detection

Mitochondrial membrane potential was detected by the JC‐1 detection kit (C2003S, Beyotime, China) according to the manufacture's protocols. Briefly, a working solution of JC‐1 (200X) was prepared by diluting the stock solution in JC‐1 staining buffer. The solution was kept protected from light. Primary cardiomyocytes were prepared in culture medium at the desired concentration and seeded into a 6‐well plate. The cardiomyocytes were incubated with JC‐1 working solution for 20 min at 37 °C in a CO_2_ incubator. The cardiomyocytes were washed three times with JC‐1 staining buffer to remove any unbound JC‐1. The cells were analyzed by fluorescence microscopy and visualized using a fluorescence microscope with filters for green (excitation 490 nm/emission 530 nm) and red (excitation 525 nm/emission 590 nm) fluorescence.

### Echocardiography Analysis

Transthoracic echocardiography was performed using the VeVo 2100 Imaging System (VisualSonics, Toronto, Canada) equipped with a 30‐MHz transducer, providing spatial resolutions of 115 µm (lateral) and 55 µm (axial). Image acquisition and analysis were conducted using Vevo LAB software (version 2.1.0). Mice were anesthetized with 1–2% isoflurane; and then, fixed on the echo pad in a supine position. The heart rates of mice were recorded and maintained at ≈500 bpm/min^−1^. 2D long‐axis M‐mode measurements were recorded at the level of the papillary muscles. The averaged interventricular septum (IVS) diastolic and systolic thickness (IVSd, IVSs), left ventricular (LV) diastolic and systolic posterior wall thickness (LVPWd, LVPWs), and LV diastolic and systolic internal dimensions (LVIDd, LVIDs) were quantified. LV fractional shortening (FS) [(LVIDd – LVIDs)/LVIDd] and LV ejection fraction (EF) [(LV Vol;d−LV Vol;s)/LV Vol;d × 100%] were calculated from these M‐mode measurements.

### Neonatal Mice Cardiomyocytes Culture

The 1–3 d neonatal C57BL/6 mice were disinfected with 75% alcohol; and then, the chest cavity was dissected open to excise the hearts. The hearts were washed three to four times with cold PBS; and then, minced into 1–2 mm^3^ pieces and digested overnight using HBSS with trypsin (1 mg mL^−1^, PH 7.2, Amresco)at 4 °C. The next day, the supernatant was discarded; and then, the pieces were redigested with collagenase II at 37 °C in a water bath for 25 min. After digestion, it was mixed well with a pipette for 50 times; and then, filtered with 70 µm cell filters. The supernatant was centrifuged at 1000 rpm for 5 min to collect cell pellets; then, resuspended in a high glucose DMEM (Gibco, 11965092) with 10% fetal bovine serum (FBS, Biological Industries) medium. Cells were seeded into a 10cm^2^ dish to be cultured for 1 h; and then, the supernatant containing unattached cardiomyocytes was transferred to a new cell container for further experiments.

### Adult Mouse Cardiac Myocyte Isolation and Culture

Primary adult cardiomyocytes (CMs) were isolated from the ventricles of wild‐type and gene‐edited male mice as described previously with some modifications.^[^ ^58^ ^]^ Briefly, mice were anesthetized and the heart was exposed before cutting the descending aorta. 7 mL EDTA buffer (130 mm NaCl, 0.5 mm Na_2_HPO_4_, 5 mm KCl, 10 mm Taurine, 10 mm HEPES, 10 mm D‐glucose, 10 mmol L^−1^ BDM, 5 mm EDTA, pH 7.8) was injected steadily into the right ventricle. Subsequently, the heart was excised and perfused with 10 mL EDTA buffer, 3 mL perfusion buffer (130 mm NaCl, 5 mm KCl, 0.5 mm Na_2_HPO_4_, 10 mm HEPES, 10 mm Taurine, 10 mm D‐glucose, 10 mmol L^−1^ BDM, 1 mm MgCl2, pH 7.8), and 30 mL pre‐warmed collagenase buffer (perfusion buffer with 0.5 mg mL^−1^ collagenase II, 0.5 mg mL^−1^ collagenase IV, and 0.05 mg mL^−1^ protease XIV). Prior to use, the entire buffer underwent filtration through a 0.22‐µm filter. The cardiac ventricles were subsequently dissociated into pieces using collagenase buffer. Digestion was stopped using 5 mL stop buffer (perfusion buffer with 5% FBS). The resulting cell suspension was filtered through a 100‐µm filter into a 50 mL tube, where it settled into a pellet through gravity. After removing the supernatant, the pellet with CMs underwent three steps of gradient calcium reintroduction and was plated on laminin coated culture dishes. The media were replaced with M199 culture media (Gibco,12340‐030) after 1 h of plating. All the cells were incubated at 37 °C with 5% CO_2_.

### Cardiomyocyte Shortening/Relengthening Assay

A SOFTEDGE MYOCAM system (IonOptix Corporation, Milton, MA, USA) was used to determine the mechanical characteristics of cardiomyocytes as previously reported.^[^ ^59^ ^]^ In brief, the isolated cardiomyocytes from *FOXM1^fl/fl^Myh6^+^* and *Myh6^+^* mice were stimulated with 0.5 Hz frequency of FHC stimulator (Brunswick, NE, USA) before being recorded. The mechanical properties including peaking shortening (% cell lengthening), maximal velocity of relengthening (+dl/dt), and maximal velocity of shortening (−dl/dt) were calculated and analyzed using IONOPTIX SOFTEDGE software.

### Mitochondrial DNA Copy Number Quantification

Mitochondrial DNA (mtDNA) and nuclear DNA were extracted from isolated cardiomyocytes or the apex of ex vivo ischemic reperfused hearts using DNeasy Blood & Tissue kit (Qiagen). After extracting, the copy number was assessed using SYBR Green dye (TaKaRa, #RR820A). The transcript level of mtDNA was reflected by mtND1: 5‐CTCTTATCCACGCTTCCGTTACG‐3 and 5‐GATGGTGGTACTCCCGCTGTA‐3. The transcript level of nDNA was reflected by M*x*1: 5‐GACATAAGGTTAGCAGCTAAAGGATCA‐3 and 5‐TCTCCGATTAACCAGGCTAGCTAT‐3.

### ATP Content Quantification

ATP content from isolated cardiomyocytes or the apex of ex vivo ischemic reperfused hearts was measured with a commercial ATP assay kit (S0027, Beyotime Institute of Biotechnology, Shanghai, China) according to the previous report.^[^ ^60^ ^]^ Briefly, isolated cardiomyocytes were lyzed and homogenized with ATP lysis buffer before being centrifuged (12 000 × *g*, 5 min) at 4 °C. 20 uL supernatant was harvested and mixed with 100 uL ATP detection working solution in a 96‐well plate. A microplate luminometer (BioTek,Synergy2) was used to measure ATP levels shown as nmol mg^−1^ protein, which was normalized to a standard curve.

### siRNA Transfection

Foxm1 specific siRNA (si‐Foxm1), MKRN1 specific siRNA(si‐MKRN1), and their negative control siRNA sequences (NC) were designed and synthesized by RiboBio Co, Ltd. The target sequences were as follows: Foxm1 siRNA: GCTATCCAGTGAAGGAATA; Mkrn1 siRNA: TTCCTATTCAGTCCATTAA. After siRNA transfection for NVCMs using Lipofectamine RNAiMAX Reagent (Invitrogen, 13778075), cells were collected 24 h later for RNA isolation and 48 h later for protein extraction. The transfection efficiency was confirmed by western blotting and quantitative real‐time PCR.

### Adenovirus Construction and Infection

For adenovirus construction, the entire coding regions of FOXM1 and MKRN1 were inserted into the pDC315 vector with an enhanced green fluorescent protein (EGFP) reporter gene (Hanbio Co. Ltd, Shanghai, China), respectively. The recombinant adenoviruses were generated by recombinating the modified pDC315 plasmids and pBHGlox E1,3Cre backbone plasmids in competent *Escherichia coli* and amplificating in HEK293T cells by using LipofectamineTM 3000 Reagent (Invitrogen, L3000001). The Ad‐GFP were used as negative controls. NVCMs were infected with the indicated adenovirus particles at a multiplicity of infection (MOI) of 100 for 6 h before replacing the medium. 48 h after infection, cells were harvested and used for bio‐analysis. All the procedures were conducted in in a biosafety environment.

### Western Blotting

The left ventricular tissue homogenates and cell lysates were homogenized in RIPA lysis buffer (P0013C, Beyotime, China) containing protease inhibitor cocktail (Thermol, 78 443, 1:100 dilution) on ice. After being centrifugated at 13 500 × *g* at 4 °C for 15 min, supernatant fractions were collected before determining the protein concentration according to the manufacturer's instruction (P0010, Beyotime, China). Equal amount of proteins were denatured and separated in 10–15% SDS‐polyacrylamide gels. Subsequently, proteins were transferred onto poly vinylidene fluoride (PVDF) membranes (Merck, #ISEQ00010). The membranes were blocked with 5% non‐fat milk for 1 h at room temperature and incubated with primary antibodies at indicated dilutions at 4 °C overnight (See Table S3, Supporting Information). Subsequently, the horseradish peroxidase (HRP)‐conjugated anti‐rabbit or anti‐mouse secondary antibodies were used at 1:4000 for 1 h at room temperature to capture the primary antibodies. Proteins were visualized using Electrochemiluminescence (ECL) substrate (Thermo, #32132) and ChemiDoc Imaging System (Bio‐Rad, CA, US). The density after normalization were quantified using NIH ImageJ software (1.50i, US).

### Quantitative Real‐Time PCR

The total RNA of cultured cells or heart tissues was homogenized and collected using RNAiso Plus (Takara, 9109) according to the manufacturer's instructions. The concentration and purity of RNA were determined by Nanodrop. 1 ug RNA with A260/A280 ratio of 1.8–2.0 was used for subsequent reverse‐transcription by PrimeScript RT Master Mix (Takara, #RR036A). SYBR Green dye (TaKaRa, #RR820A) and specific gene primers (Table S1, Supporting Information) were used for quantitative real‐time polymerase chain reaction (qRT‐PCR) on CFX96 real‐time PCR System (Bio‐Rad Laboratories, Inc., CA, USA) according to the recommended thermal cycling conditions. The gene expression was normalized to β‐actin using the standard 2^−ΔΔCT^ method.

### Electron Microscopy

The transmission electron microscopy was conducted as follows. After being harvested, heart samples were cut into 1–2 mm piece before being fixed in electron microscopy fixative (Shanghai ribiology technology, China) for at least 3 h at 4 °C. Samples were then washed three times for 15 min before being fixed in 1% osmiumtetroxid (Science Services) in 0.1 m cacodylic acid at room temperature for 2 h and washed again four times for 15 min in 0.1 m PBS buffer. After dehydration in ethanol from 50% to 100%, the tissue was embedded in a mixture of Epon 812 fixative (90529‐77‐4, SPI, USA) and acetone overnight before polymerization. Fixed tissue was cut in 70 nm sections using ultramicrotome (UC7, Leica, USA) and contrasted with 1,5% uranylacetate aqueous solution 15 min at 37 °C. After being washed five times, incubated in lead citrate for 4 min, and washed again, the images were acquired and analyzed under transmission electron microscopy (Tecnai G2 20 TWIN, FEI, USA).

### Pig AAV Transfer and Ischemia–Reperfusion Model

All pig experiments were approved by the Animal Care and Use Committee at Zhongshan Hospital, Fudan University, China and conducted according to the previous report.^[^ ^61^ ^]^ Briefly, pigs were anesthetized using 6 mg kg^−1^ zolazepam and tiletamine (Virbac) by intramuscular injection and were maintained with isoflurane by inhalation. After a 7‐F femoral artery sheath was engaged, a coronary artery guiding catheter was selectively placed into the left main coronary artery ostium and AAV9‐FOXM1 or control vector was delivered through the catheter. 1 month after transfer, the surviving pigs were anesthetized, punctured through the left femoral artery, and a percutaneous transluminal coronary angioplasty balloon (Woten, 3.0 mm × 15 mm) was placed distal to the bifurcation of the first diagonal branch of LAD, which was later inflated with 8 atm to occlude LAD. The localization of the balloon was confirmed by coronary angiogram. 60 min after occlusion‐induced ischemia, the balloon was deflated and the initiation of reperfusion was recorded angiographically. The measurement of cardiac function by MRI and infarct size by histological analysis were performed 2 months after surgery. TTC staining and picosirius staining were performed as previously described.

### MRI Measurement Image Acquisition and Analysis

The cardiac function of pigs was examined by magnetic resonance imaging (MRI) using a clinical 3T magnetic resonance scanner (Skyra; Siemens, Erlangen, Germany) according to the previous studies with minor modifications.^[^ ^62^ ^]^ Briefly, 2 months after IR surgery, pigs were anesthetized with 6 mg kg^−1^ zolazepam and tiletamine (Virbac) before undergoing MRI examination with cines and late gadolinium enhancement (LGE) imaging sequences (gadoteric acid was given at 0.02 mmol kg^−1^ intravenous). The heart was scanned in coronary views (field of view, 264 × 320 mm^2^; section thickness, 8 mm; image acquisition matrix, 240 × 158; bandwidth, 945 Hz pixel^−1^; repetition time, 31.14 ms; echo time, 1.52 ms; flip angle 41°), and transverse views (field of view, 264 × 320 mm^2^; section thickness, 8 mm; image acquisition matrix, 240 × 158; bandwidth, 945 Hz pixel^−1^; repetition time, 31.14 ms; echo time, 1.52 ms; flip angle 41°). ECG, heart rates, and respiration were all monitored during the examination. The cardiac MRI image analysis was performed using the research software package (cvi42 v5.9.4, Circle Cardiovascular Imaging Inc) blindedly, which was supervised by independent radiologists. Ejection fraction (EF) was calculated and analyzed according to the cine MRI images. Left ventricular end diastolic volume (LVEDV) and left ventricular end systolic volume (LVESV) were analyzed according to the LGE MRI images.

### Histopathology Analysis

The hearts of the mice were perfused with cold PBS and harvested. After fixing in 4% paraformaldehyde for 48 h, the hearts were embedded in paraffin. For hematoxylin and eosin (H&E) staining, heart sections were longitudinally obtained and the staining was performed according to the manufacturer's protocols of the H&E buffer (Servicebio, G1005). For picrosirius red (PSR) staining, the heart was transversely sectioned at 6 µm intervals, and the slides were incubated with Sirius Red solution (Solarbio, G1018) at room temperature for 1 h. The fibrotic area in each field was quantified using Image‐Pro Plus software. For quantification, total fibrosis was averaged from two to three fields per section and three heart sections per mouse. Each group contained ten mice.

### Immunofluorescence Staining

Hearts were embedded in paraffin and sliced into 6 µm serial sections. Adult mice cardiomyocytes and heart sections were fixed in 4% paraformaldehyde, blocked with 3% BSA, and permeabilized with 0.3% Triton‐X100. The cells and heart sections were incubated with primary antibody against α‐actinin (Sigma–Aldrich, A5044, 1:200 dilution), FOXM1 (CST, 20459, 1:200), or Tomm20 (Abcam, ab186735, 1:200) according to the indicated groups at 4 °C overnight. Next day, the cells and heart sections were then incubated with Alexa Fluor Plus 647 Goat anti‐mouse IgG (H+L) Highly Cross‐Adsorbed Secondary Antibody (Invitrogen, A32728, 1:500 dilution) and Alexa Fluor Plus 488 Goat anti‐Rabbit IgG (H+L) Highly Cross‐Adsorbed Secondary Antibody (Invitrogen, A32731, 1:500 dilution) at room temperature for 1 h away from light. After washing three times for 5 min, 4,6‐Diamidino‐2‐phenylindole (DAPI) was used to stain the nuclei for another 10 min in the dark. The immunofluorescence staining was photographed on a Zeiss fluorescence microscope, and the fluorescence intensity was quantified by Image‐Pro Plus software (version 6.0).

For TUNEL staining, the One Step TUNEL Apoptosis Assay Kit (Beyotime, Shanghai, China) was used according to the manufacturer's instructions. Briefly, the samples were fixed and then stained with the TUNEL regent. After counterstaining with DAPI, the samples were detected by a Zeiss fluorescence microscope.

For wheat germ agglutinin (WGA) staining, the heart sections were incubated with FITC‐conjugated wheat germ agglutinin (WGA, W11261, Invitrogen) for 15 min at room temperature. The cardiomyocyte cross‐sectional areas were visualized using Zeiss fluorescence microscope with captured images. All analysis was conducted by an investigator blind to the group information.

### Co‐Immunoprecipitation

The co‐immunoprecipitation was performed according to the instructions of Pierce Classic IP Kit (Thermo Scientific, 26146) with some modifications. Briefly, isolated adult mice cardiomyocytes or cultured HEK 293T cells were washed with PBS and lyzed with ice cold IP Lysis/Wash Buffer. After centrifugation at 13 000 × *g* for 10 min, the supernatant was transferred to a new tube for protein concentration determination. Then, the lysates were preincubated with Control Agarose Resin slurry for 1 h and incubated with IgG (CST, 3900) control, MKRN1 antibody (abcam, ab72054), or Flag antibody (CST, 14793) overnight at 4 °C with rotation. Next day, the Pierce Protein A/G Agarose was added to the lysates incubating for 1 h at 4 °C, and agarose–antibody‐antigen complexes were collected using a pierce spin column. After three times washing in IP Lysis/Wash Buffer and once washing in 1X Conditioning Buffer, proteins were separated from beads by resuspending in 50 µL of 2 × loading buffer and boiling at 95 °C for 10 min. Samples were then analyzed by immunoblotting. The Clean‐Blot IP Detection Kit (Thermo Scientific, 21232) was used to detect target proteins without interference from heavy and light chains of IgGs.

### Dual Luciferase Assays

HEK 293T cells were seeded into 24‐well plates and transfected with plasmids expressing Flag‐Foxm1 (0.5 µg per well), Mkrn1 luciferase reporter (firefly luciferase, 0.5 µg per well), and Renilla luciferase plasmids (0.01 µg per well) using Lipofectamine 3000 Reagent (Invitrogen, L3000001). After 24 h cultivation, the cells were harvested and the luciferase activity was measured using a dual luciferase reporter assay system (Promega, USA) following the standard protocol. Firefly luciferase activities were normalized by the Renilla luciferase activities.

### ChIP‐qPCR

Chromatin immunoprecipitation, followed by qPCR (ChIP‐qPCR), was performed according to the standard crosslinking ChIP protocol previously reported with modifications.^[^ ^63^ ^]^ Briefly, the HEK 293T cells were incubated with 1% formaldehyde for 10 min at room temperature before being neutralized by adding 1X glycine (CST, 7005). Then, cells were washed with cold PBS three times and collected by centrifugation at 1500 rpm. Subsequently, cells were lyzed using prepared Nuclei Isolation Buffer (20 mm HEPES pH 7.5, 10 mm KCl, 1 mm EDTA, 0.2% NP40, 10% Glycerol, 1 × Protease Inhibitor Cocktail [PIC; Roche]), and sonicated at procedures of 80% amplitude, pulse for 5 s on and 5 s off for a total sonication “on” time of 10 min of elapsed time (Qsonica Q125, America). After sonication, soluble chromatin was acquired by harvesting supernatants after centrifugation at 21 000 × *g* for 10 min at 4 °C. DNA gel electrophoresis was performed to ensure suitable chromatin fragmentation status. Soluble chromatin was then incubated with protein G magnetic beads (CST, 9006) for pre‐cleaning and further immunoprecipitated with IgG (CST, 3900) control or Flag (CST, 14793) antibody on a rotator at 4 °C overnight. The next day, protein G magnetic beads were added into the chromatin‐protein‐antibody complex and rotated at 4° for 4 h. After being pelleted by magnetic stands, beads were washed with RIPA Buffer (50 mm HEPES pH 8.0, 1 mm EDTA, 1% NP40, 0.7% sodium deoxycholate, 1% TritonX‐100, coaktail) and de‐crosslinked with SDS elution buffer (50 mm Tris Cl pH 8.0, 10 mm EDTA, 1% SDS) at 65  °C overnight. The de‐crosslinked sample was incubated with RNase A (Invitrogen, EN0531) and proteinase K (Beyotime, ST535) simultaneously to remove RNA and protein. Then, the eluted DNA was purified by QIAquick PCR Purification kit (QIAGEN, 28104), and qPCR was performed. The used primers for qPCR are listed below:

Human LKB1, forward: 5′‐TCGACCTGGGAACTGCAGTA‐3′
Human LKB1, reverse: 5′‐GCATTGGCAATAGATCCAAC‐3′;
Human MKRN1, forward: 5′‐AAGTGCTGGGATTACAGGCG ‐3′
Human MKRN1, reverse: 5′‐CAGCCCAATCCTGGAAGAGG‐3′;

### RNA‐Sequencing and Data Analysis

Total RNA was extracted from cardiac tissue of mice using the miRNA mirVana Isolation Kit (Ambion) according to the manufacturer's protocol. RNA integrity was assessed using an Agilent 2100 Bioanalyzer (Agilent Technologies, Santa Clara, CA), followed by analysis of samples with an RNA Integrity Number (RIN) of 7 or higher. The construction of libraries was completed using the TruSeq Stranded mRNA LTSample Prep Kit from Illumina (San Diego, CA, USA), following the manufacturer's instructions. Subsequently, these libraries underwent sequencing on the Illumina sequencing platform (HiSeqTM 2500 or Illumina HiSeq X Ten) to produce 125 bp/150 bp paired‐end reads. To obtain clean reads, the raw data (raw reads) were processed using Trimmomatic, which involved the removal of reads containing poly‐N and low‐quality reads before mapping the clean reads to the reference genome by hisat2. Cufflinks were used to obtain FPKM value of each gene and read counts were calculated using htseq‐count. DESeq (2012) R package functions estimateSizeFactors and nbinomTest were utilized to identify differential expressed genes (DEGs) with a pre‐set threshold of FDR‐corrected q‐value < 0.05 and foldChange < 1/1.5 or foldChange >1.5. The R package of “pheatmap,” “clusterProfiler,” “GOplot,” and “enrichplot” were utilized for hierarchical cluster analysis, GO enrichment analysis, KEGG pathway analysis, and GSEA enrichment analysis of DEGs, respectively.

### Mass Spectrometry Analysis

The isolated CMs were denatured for DIA analysis using a 2% SDS buffer containing 50 mm DTT for 20 min at room temperature. The denatured cells were then boiled at 100 °C for 5 min. To alkylate the protein sample, a final concentration of 10 mm iodoacetamide (IAA) was added and left to react for 15 min at room temperature in the dark. Subsequently, six times volume of pre‐cooled acetone was added to precipitate the proteins overnight at −20 °C. The precipitated proteins were collected using centrifuger at at 8000 × *g* and 4 °C for 15 min before being digested using sequencing grade modified trypsin (Promega) at a protein‐to‐enzyme ratio of 50:1 at 37 °C overnight. Tryptic peptides were treated with 1% trifluoroacetic acid (TFA) and purified using C18 Ziptips. The peptide was eluted with 0.1% TFA in 50–70% acetonitrile, which was then lyophilized using a SpeedVac (ThermoSavant). For ubiquitinome, Ubiquitin Remnant Motif (K‐ε‐GG) Kit Immunoaffinity Beads (5562, CST, USA) were used to capture and purified the ubiquitinated peptides. For proteome, whole peptides were digested and further separated by high pH reverse phase chromatography, and each spectral library was constructed. Digests were frozen with SpeedVac (ThermoSavant) and resuspended in 5% ACN with 0.05 m ammonium. The digested peptides underwent high pH reversed phase separation using a Dionex UHPLC (Thermo Scientific, Waltham, Massachusetts, United States) equipped with a 2.1 × 150 mm ethylene bridged hybrid (BEH) C18 3 µm column (Waters) at a temperature of 40 °C, with a flow rate of 0.2 mL min^−1^ and a 60‐min ACN gradient (≈5–30%) in 5 mm ammonium format (pH 10).

### Ubiquitinated Peptide Enrichment

The total protein in the sample was extracted, and a portion was taken out for protein concentration determination and SDS‐PAGE detection. The other part was subjected to trypsin digestion, peptide desalting, and immunoaffinity purification of the modified peptides. Finally, the samples were subjected to LC‐MS/MS analysis and data analysis. The results of maxquant library search were statistically analyzed and screened for ubiquitinated plausible proteins/peptides/sites. Functional analysis of the proteins corresponding to the screened significant ubiquitination sites was also performed, including GO analysis, pathway analysis, and protein interactions. It also included site‐motif analysis, differential site‐expression pattern, and Venn analysis.

### Statistical Analysis

Data are presented as mean ± standard deviation (SD) for statistical analysis. Shapiro–Wilk test was applied to assess normality of the distribution of data. Before *t*‐test and analysis of variance (ANOVA), homogeneity was tested among variances by F test (for two groups) and Brown–Forsythe test (for > two groups). For data of two groups with normal distribution and equal variances, statistical differences were analyzed using two‐tailed Student's *t*‐test. For data of > two groups with normal distribution and equal variances, statistical differences were analyzed using one‐way ANOVA (one variable involved) and two‐way ANOVA (two variables involved) with Bonferroni multiple comparison test. Statistical differences of data with unequal variance were evaluated by Welch's *t*‐test (for two groups) or Welch's ANOVA with Dunnett's T3 multiple comparison test (for > two groups). Two‐sided *P* values less than 0.05 were considered as statistically significant. All statistical analyses were performed with Prism software (GraphPad prism for windows, version 9.0, Nashville, USA).

## Conflict of Interest

The authors declare no conflict of interest.

## Author Contributions

S.S., X.Z., Z.H., and Z.P. contributed equally to the study. A.S. and J.G. conceived and designed the study. S.S., X.Z., Z.H., Z.P., L.Z., F.C., T.W., M.L., C.L., Y.S., J.G., H.L., X.W., X.Z., and X.C. performed the experiments and analyzed the data. S.S. and X.Z. drafted the manuscript. A.S. and J.G. critically revised important intellectual content within the manuscript. All the authors provided final approval for the version of the manuscript to be published.

## Supporting information



Supporting Information

Supporting Information

Supporting Information

Supporting Information

## Data Availability

The data that support the findings of this study are available from the corresponding author upon reasonable request.
