# Supplementary material for: FOXM1 Protects Against Myocardial Ischemia‐Reperfusion Injury in Rodent and Porcine Models by Suppressing MKRN1‐Dependent LKB1 Ubiquitination
Source: Adv Sci (Weinh). 2025 Jun 23;12(35):e08673. doi: 10.1002/advs.202508673 (PMC12463072; doi:10.1002/advs.202508673)
Supplement: Supplementary file 1 — Supporting Information [file ADVS-12-e08673-s003.docx]

**SUPPLEMENTARY MATERIAL**

**Supplementary figure 1. The construction of cardiomyocyte specific FOXM1 deletion mice.**

**A.** Schematic diagram for generating cardiomyocyte-specific tamoxifen-inducible FOXM1 knockout mice. **B.** The DNA agarose gel electrophoresis showing genotyping results of FOXM1-cKO mice. **C-D.** The mRNA expression levels (n=6) **(C)** and protein levels (n=3) **(D)** of FOXM1 in heart samples of *Foxm1^fl/fl^Myh6*^+^ and *Myh6*^+^ mice. **E-F.** The mRNA expression levels (n=6) **(E)** and protein levels (n=3) **(F)** of FOXM1 in cardiomyocytes isolated from *Foxm1^fl/fl^Myh6*^+^ and *Myh6*^+^ mice.

For all statistical plots, the data are presented as mean ± SD. C-D by two-tailed unpaired Student’s t-test. E-F by Welch’s t-test.

**Supplementary figure 2. Cardiac mitochondrial dysfunction induced by FOXM1 deficiency.**

**A.** Volcano plot showing the gene expression of significantly upregulated and downregulated genes based on RNA-seq data in heart samples of *Myh6*^+^ and *Foxm1^fl/fl^Myh6^+^* mice. **B-C.** GO term enrichment analysis of upregulated (**B**) and downregulated (**C**) genes in heart samples of *Myh6*^+^ and *Foxm1^fl/fl^Myh6^+^* mice show the top 8 biological process based on RNA-seq data. **D.** Heatmaps of differentially expressed genes (DEGs) in heart samples of *Myh6*^+^ and *Foxm1^fl/fl^Myh6^+^* mice based on RNA-seq data. **E-F.** qRT-PCR analyses of mitochondrial complex **(E)** and ECM genes **(F)** mRNA levels in heart samples of *Myh6*^+^ and *Foxm1^fl/fl^Myh6^+^* mice (n=3). **G.** Western blot of mitochondrial complex proteins in heart samples of *Myh6*^+^ and *Foxm1^fl/fl^Myh6^+^* mice (n=3). **H.** Representative electron microscopy images and quantitative analysis of mitochondrial in heart samples of *Myh6*^+^ and *Foxm1^fl/fl^Myh6^+^* mice (n=10; scale bar=5 μm in upper panel and 2 μm in lower panel). For all statistical plots, the data are presented as mean ± SD. E and F by two-tailed unpaired Student’s t-test. H by Welch’s t test.

**Supplementary figure 3. FOXM1 regulates mitochondrial function through AMPK signaling pathway.**

**A.** Seahorse real-time traces and averaged data for mitochondrial oxygen consumption rate (OCR) were measured in cardiomyocytes isolated from *Myh6*^+^ and *Foxm1^fl/fl^Myh6^+^* mice in the presence or abcense of AMPK activator A769662 (100μM) using Seahorse XFe24 Analyzer (n=10). **B.** Representative confocal images of mitochondrial transmembrane potential (Δψm) labelled by JC-1 monomer (green, marking depolarization) and JC-1 aggregate (red, marking hyperpolarization) in cardiomyocytes isolated from *Myh6*^+^ and *Foxm1^fl/fl^Myh6^+^* mice in the presence or abcense of AMPK activator A769662 (100μM) (n=10; scale bar=20 μm). C. Western blot and quantification of p-AMPK and AMPK in cultured NVCMs infected with adenovirus expressing FOXM1 or NC and then treated with CAMKK2 inhibitor STO-609 (25μM) (n=4). For all statistical plots, the data are presented as mean ± SD. A and C by one-way ANOVA with Bonferroni multiple comparison test.

**Supplementary figure 4. Quantitative ubiquitinome analysis in cardiomyocytes isolated from *Myh6*^+^ and *Foxm1*^fl/fl^*Myh6*^+^ mice**

**A.** Identification of ubiquitinated proteins, ubiquitinated peptides and ubiquitinated sites. **B.** Identification of the number of proteins corresponding to different molecular weight. **C.** Identification of the distribution of ubiquitination sites corresponding to ubiquitinated proteins. **D.** Identification of the percentage of peptides corresponding to each ubiquitinated site in the total ubiquitinated peptides. **E.** Volcano plot of the ubiquitin sites changes in response to Foxm1 deficiency. Blue dots in the graph indicate down-regulated ubiquitin sites; red dots are up-regulated sites; and gray dots are non-significant differentially sites. **F.** Identification of ubiquitinated differential site motifs. The horizontal coordinate indicates the position of the amino acid in the Sequence window, and the vertical coordinate indicates the relative frequency of the amino acid at each site.

**Supplementary figure 5. Identifying the expression of MINDY2, MKRN1 and RBX1 in response to *Foxm1* deficiency.**

**A.** qRT-PCR analyses of mRNA levels of *Mindy2*, *Mkrn1* and *Rbx1* in cardiac tissues from *Foxm1^fl/fl^Myh6*^+^ and *Myh6*^+^ mice (n=6). **B.** Western blot and quantification of MKRN1 and RBX1 in cardiac tissues from *Foxm1^fl/fl^Myh6*^+^ and *Myh6*^+^ mice (n=3). For all statistical plots, the data are presented as mean ± SD. A, B by two-tailed unpaired Student’s t test.

**Supplementary Figure 6. The construction of cardiomyocyte specific MKRN1 deletion mice.**

**A.** Schematic diagram for generating Mkrn1 cKO mice. **B.** The DNA agarose gel electrophoresis showing genotyping results of Mkrn1-cKO mice. **C.** qRT-PCR analyses of mRNA levels of *Mkrn1* and *Foxm1* in cardiac tissues from *Foxm1/ Mkrn1^fl/fl^Myh6^+^* and *Myh6*^+^ mice (n=6). **D.** Western blot and quantification of MKRN1 and FOXM1 in cardiac tissues from *Foxm1/ Mkrn1^fl/fl^Myh6^+^* and *Myh6*^+^ mice (n=3). **E.** qRT-PCR analyses of the mRNA levels of *Mkrn1* and *Foxm1* in primary cardiomyocytes from *Foxm1/ Mkrn1^fl/fl^Myh6^+^* and *Myh6*^+^ mice (n=6). **F.** Western blot and quantification of MKRN1 and FOXM1 in cardiomyocytes isolated from *Foxm1/ Mkrn1^fl/fl^Myh6^+^* and *Myh6*^+^ mice (n=3). For all statistical plots, the data are presented as mean ± SD. C-D by two-tailed unpaired Student’s t-test. E-F by Welch’s t-test.

**Supplementary Figure 7. Mkrn1 Knockout attenuates Foxm1 deficiency-induced cardiac remodeling and cardiac dysfunction.**

**A.** M-mode echocardiography and echocardiographic parameters of *Myh6^+^*, *Mkrn1^fl/fl^Myh6^+^*, *Foxm1^fl/fl^Myh6^+^*, and *Foxm1/ Mkrn1^fl/fl^Myh6^+^* mice (n=10). IVS, interventricular septum. **B.** Heart weight to body weight ratio (HW/BW, mg/g) in *Myh6^+^*, *Mkrn1^fl/fl^Myh6^+^*, *Foxm1^fl/fl^Myh6^+^*, and *Foxm1/ Mkrn1^fl/fl^Myh6^+^* mice (n=10). **C.** Representative wheat germ agglutinin (WGA) staining in *Myh6^+^*, *Mkrn1^fl/fl^Myh6^+^*, *Foxm1^fl/fl^Myh6^+^*, and *Foxm1/ Mkrn1^fl/fl^Myh6^+^* mice. (n=10, scale bar = 20 μm). **D.** Representative picrosirius red staining of *Myh6^+^*, *Mkrn1^fl/fl^Myh6^+^*, *Foxm1^fl/fl^Myh6^+^*, and *Foxm1/ Mkrn1^fl/fl^Myh6^+^* mice (n=10, scale bar = 50 μm). **E.** Western blot and quantification of Fibronectin, Collagen I, ANP and BNP in cardiac tissues of mice from indicated groups. *Myh6^+^*, *Mkrn1^fl/fl^Myh6^+^ (M^fl/fl^M^+^)*, *Foxm1^fl/fl^Myh6^+^(F^fl/fl^M^+^)*, and *Foxm1/ Mkrn1^fl/fl^Myh6^+^(F/M^fl/fl^M^+^)* mice (n=4). **F**. Representative micrograph and quantitative analysis of intracellular reactive oxygen species (ROS) in cardiomyocytes isolated from *Foxm1^fl/fl^Myh6^+^* and *Foxm1/Mkrn1^fl/fl^Myh6^+^* mice (n=10). **G.** Western blot and quantification of SDHA, UQCRC1, COX5A and ATP5A in cardiac tissues of mice from indicated groups. *Myh6^+^*, *Mkrn1^fl/fl^Myh6^+^ (M^fl/fl^M^+^)*, *Foxm1^fl/fl^Myh6^+^(F^fl/fl^M^+^)*, and *Foxm1/ Mkrn1^fl/fl^Myh6^+^(F/M^fl/fl^M^+^)* mice (n=4). For all statistical plots, the data are presented as mean ± SD. A-E and G by two-way ANOVA with Bonferroni multiple comparison test. F by two-tailed unpaired Student’s t-test.


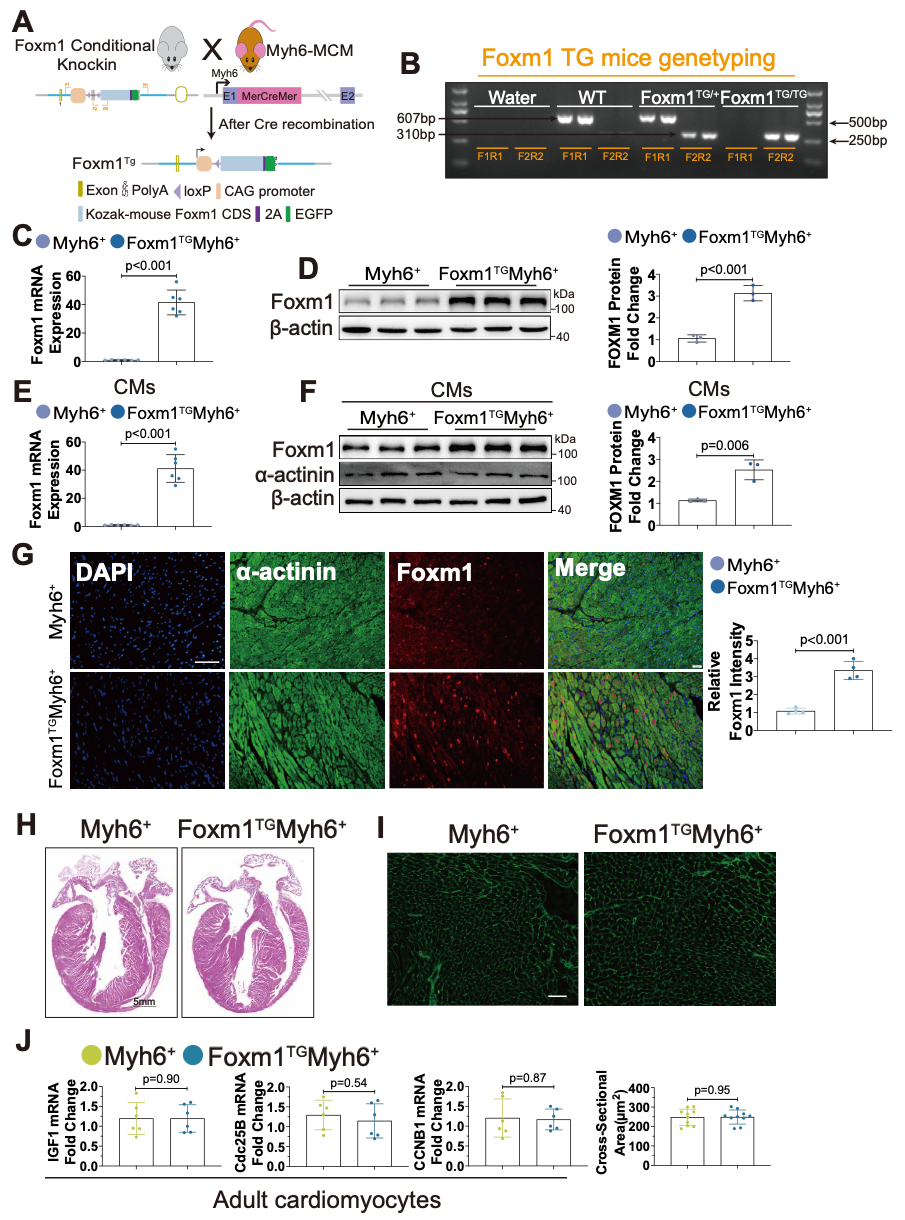


**Supplementary figure 8. The construction of cardiomyocyte specific Foxm1 overexpression mice.**

**A.** Schematic diagram for generating cardiomyocyte-specific tamoxifen-inducible Foxm1 overexpression mice. FOXM1^Tg^, FOXM1 conditional transgene mice. **B.** The DNA agarose gel electrophoresis showing genotyping results of Foxm1-Tg mice. **C.** qRT-PCR analyses of mRNA levels of FOXM1 in heart samples of *Foxm1^TG^Myh6*^+^ and *Myh6*^+^ mice (n=6). **D.** Western blot and quantification of FOXM1 in heart samples of *Foxm1^TG^Myh6*^+^ and *Myh6*^+^ mice (n=3). **E.** qRT-PCR analyses of mRNA levels of FOXM1 in cardiomyocytes isolated from *Foxm1^TG^Myh6*^+^ and *Myh6*^+^ mice (n=6). **F.** Western blot and quantification of FOXM1 in cardiomyocytes isolated from *Foxm1^TG^Myh6*^+^ and *Myh6*^+^ mice (n=3). **G.** Immunofluorescence of DAPI (4’,6-diamidino-2- phenylindole; blue), α-actinin (green) and FOXM1 (red) in myocardium of *Foxm1^TG^Myh6*^+^ and *Myh6*^+^ mice (n=4, scale bar = 20 μm). **H.** Heart sections from *Myh6^+^* and *Foxm1*^TG^*Myh6*^+^ mice were stained with hematoxylin and eosin to show whole-heart gross images (n=10; scale bar=5 mm). **I.** Heart sections from *Myh6^+^*and *Foxm1*^TG^*Myh6*^+^ mice were stained with WGA to demarcate the cell boundaries (n=10; scale bar=20 μm). **J.** qRT-PCR analyses of mRNA levels of IGF1, Cdc25B and CCNB1 in cardiomyocytes isolated from *Foxm1^TG^Myh6*^+^ and *Myh6*^+^ mice (n=6). For all statistical plots, the data are presented as mean ± SD. C-G by Welch's t-test. I-J by two-tailed unpaired Student’s t-test.

**Supplementary figure 9. Foxm1 overexpression ameliorates I/R-induced cardiac remodeling and cardiac dysfunction.**

**A.** Western blot and quantification of NDUFS3, SDHA, UQCRC1, COX5A and ATP5A in cardiac tissues of mice from indicated groups (n=4). **B**. Representative micrograph and quantitative analysis of intracellular reactive oxygen species (ROS) in cardiomyocytes isolated from *Myh6*^+^ and *Foxm1*^TG^*Myh6*^+^ mice 24 hours after I/R surgery (n=10). **C.** M-mode echocardiography and FS of *Foxm1^TG^Myh6*^+^ and *Myh6*^+^ mice 4 weeks after IR surgery (n=10). **D.** Western blot and quantification of Fibronectin, Collagen I, ANP and BNP in cardiac tissues of mice from indicated groups (n=4). For all statistical plots, the data are presented as mean ± SD. A and C-D by two-way ANOVA with Bonferroni multiple comparison test. B by two-tailed unpaired Student’s t-test. F^TG^M^+^, *Foxm^TG^Myh6^+^*. FS, fractional shortening.

**Supplementary figure 10. Overexpressing FOXM1 mitigates I/R-induced heart failure in pigs.**

**A.** Digital subtraction angiography (DSA) showing the left anterior descending artery before and after balloon occlusion. **B.** Left panel: Western blot of FOXM1 in kidney, lung, liver and heart tissues of pigs subjected to AAV9-Foxm1 administration. Right panel: Western blot of FOXM1 in different cardiac segments dominated by left anterior descending artery (LAD), left circumflex branch (LCX) and right coronary artery (RCA) of pigs after AAV9-Foxm1 or AAV9-NC administration. **C.** Western blot and quantification of Fibronectin, Collagen I, ANP and BNP in cardiac tissues of pigs at 2 months after I/R surgery with AAV9-Foxm1 or AAV9-NC administration (n=3). **D.** Western blot of FOXM1 in cardiac tissues of pigs subjected to AAV9-Foxm1 or AAV9-NC administration for 6 months. **E.** Serum concentrations of alanine aminotransferase (ALT), alkaline phosphatase (ALP), creatinine (CREA), and creatine kinase (CK) were measured in pigs prior to AAV9-FOXM1 administration and at six months post-administration. For all statistical plots, the data are presented as mean ± SD. C by one-way ANOVA with Bonferroni multiple comparison test. E by two-tailed unpaired Student’s t-test.

**Supplementary Tables**

**Supplementary Table 1.** Table of sequences and downstream primers for genes analyzed by qRT-PCR(h-human, m-mouse, p-pig).

| Gene | Forward Primer | Reverse Primer |
| --- | --- | --- |
| Foxm1(h) | 5’-gatctgcgagattttggtacac-3’ | 5’-ctgcagaagaaagaggagctat-3’ |
| Foxm1(p) | 5’-ctgcccaacaagagcctagtcaag-3’ | 5’-ccattgctgccactctcctttcc-3’ |
| Foxc2(m) | 5’-catccgccacaacctgtcactc-3’ | 5’-ccttgtccttgggcacatccttc-3’ |
| β-actin (h) | 5’-aggattcctatgtgggcgac-3’ | 5’-atagcacagcctggatagcaa-3’ |
| β-actin (m) | 5’-tatgctctccctcacgccatcc-3’ | 5’-gtcacgcacgatttccctctcag-3’ |
| β-actin (p) | 5’-gacatggagaagatctggca-3’ | 5’-gtctcgaacatgatctgggt-3’ |
| Ndufa2 (m) | 5’-cagccagggtgtgagggatttc-3’ | 5’-ggatcagaatgggcaggttggg-3’ |
| Ndufa3 (m) | 5’-gctggtggtgtccttctctgtc-3’ | 5’-atcaggcatgttcccgtcatctc-3’ |
| Sdha (m) | 5’-tgtggacatcaagactggcaagg-3’ | 5’-aggagcggatagcaggaggtac-3’ |
| Sdhc (m) | 5’-ggaaccacagctaaggaggagatg-3’ | 5’-cggacagtgccataggaagagac-3’ |
| Uqcc1 (m) | 5’-cgagctgcgtggagaagactg-3’ | 5’-ttcctgcttcattcggactagacac-3’ |
| Uqcrq (m) | 5’-ctatttcagcaaaggcatccccaac-3’ | 5’-gccccatgtgtagatcaggtagac-3’ |
| Cox6c (m) | 5’-gagttgccgctgcctataagtttg-3’ | 5’-gataccagccttcctcatctcttcg-3’ |
| Cox5a (m) | 5’-attgatgcctgggaattgcgtaaag-3’ | 5’-ccttaacaacctccaagatgcgaac-3’ |
| Atp1a2 (m) | 5’-cagcggaaggtggtggagttc-3’ | 5’-actgagttgcgacgggtcttg-3’ |
| Atp5c1 (m) | 5’-aataccattgcgactgctgagacc-3’ | 5’-tctgctcactggtggtggactc-3’ |
| Col1a1 (m) | 5’-acaggcgaacaaggtgacagag-3’ | 5’-aggagaaccaggagaaccaggag-3’ |
| Col3a1 (m) | 5’-acgaggtgacaaaggtgaaactgg-3’ | 5’-agaacctggaggacctggattgc-3’ |
| Col5a1 (m) | 5’-cctactcagaagccagtggaagc-3’ | 5’-gtcctccttgtcagccgtgtc-3’ |
| Fn1 (m) | 5’-caccgacgaagagcccttacag-3’ | 5’-ccttgtgcctcctctggttctg-3’ |
| Lox (m) | 5’-acgatttccgcaaagagtgaagaac-3’ | 5’-tggcatcaagcaggtcatagtgg-3’ |
| Postn (m) | 5’-gccactaccactcagcactactc-3’ | 5’-ggttctcccaagcctcgttactc-3’ |
| Nppa (m) | 5’-aagaacctgctagaccacctggag-3’ | 5’-tgcttcctcagtctgctcactcag-3’ |
| Nppb (m) | 5’-ggaagtcctagccagtctccagag-3’ | 5’-gccttggtccttcaagagctgtc-3’ |
| Tgfb1 (m) | 5’-caacaattcctggcgttaccttgg-3’ | 5’-tgtattccgtctccttggttcagc-3’ |
| Tgfbr1 (m) | 5’-ttgctggtccagtctgcttcg-3’ | 5’-gtggtgaatgacagtgcggttatg-3’ |
| Lkb1 (m) | 5’-acaccttcatccaccgcatcg-3’ | 5’-gtccagcacctccttcaccttg-3’ |
| Mindy2(m) | 5’-caggctcaggctgttgtgactac-3’ | 5’-tttcccgaggttccttgcgtttc-3’ |
| Mkrn1(m) | 5’-accatccctcgctgcttcctc-3’ | 5’-ccagtcctctgaacctgctcctac-3’ |
| Rbx1(m) | 5’-gcggcggcgatggatgtg-3’ | 5’-acaatgtcccaggcccagagg-3’ |

**Supplementary Table 2.** Clinical characteristics of patients with Normal or ICM.

| Normal (n=7) ICM (n=7) p |
| --- |
| Age of diagnosis (years) 51.32±4.63 61.21±4.21 <0.001  Female n (%) 4 (57.1%) 4 (57.1%) >0.999  BMI 22.34±3.03 23.01±2.27 0.625 |
| Echocardiographic parameters  LVEDD (mm) 42.24±4.26 63.76±6.56 <0.001  LVESD (mm) 33.32±3.28 41.73±7.04 0.008  LVEF (%) 59.56±5.46 34±5.06 <0.001 |
| Diseases  Hypertension (%) 2 (29%) 6 (86%) 0.132  Atrial fibrillation (%) 1 (14%) 2 (29%) >0.999  Valvular heart disease (%) 0 (0%) 1 (14%) >0.999  Diabetes (%) 2 (29%) 4 (57%) 0.608  Hyperlipemia (%) 2(29%) 6 (86%) 0.132  Medical therapy prior to the surgery  Anticoagulant n (%) 1 (14%) 2(29%) >0.999  Antiplatelet n (%) 2 (29%) 7 (100%) 0.007  Statin n (%) 3 (43%) 7 (100%) 0.026  Diuretics n (%) 0 (0%) 6 (86%) 0.007  β-blockers n (%) 2 (29%) 5 (71%) 0.305  ACEI/ARB n (%) 1 (14%) 6 (86%) 0.041 |

Data are expressed as mean ± SD. Data of Ages, BMI and Echocardiographic parameters were analyzed using two-tailed unpaired Student’s t-test. Others were analyzed using Fisher exact test. ICM, Ischemic cardiomyopathy; BMI, body mass index; LVEDD, left ventricular end diastolic diameter; LVESD, left ventricular end systolic diameter; LVEF, left ventricular ejection fraction; ACEI, angiotensin-converting enzyme inhibitor; ARB, angiotensin receptor blocker

**Supplementary Table 3.** The information of antibodies.

| Name | Vender | Cat |
| --- | --- | --- |
| FOXM1 | Abcam | ab180710 |
| FOXM1 | CST | 20459 |
| α-actinin | Sigma-Aldrich | A5044 |
| TOMM20 | abcam | ab186735 |
| ANP | Proteintech | 27426-1-AP |
| BNP | Abcam | ab239510 |
| β-actin | CST | 4970 |
| FIBRONECTIN | Abcam | ab268020 |
| COLLAGEN I | Abcam | ab260043 |
| MT-ND1 | Abclonal | A17967 |
| SDHA | Abclonal | A13852 |
| UQCRC1 | Abclonal | A3339 |
| COX5A | Abclonal | A6437 |
| ATP5A | Abclonal | A5884 |
| VDAC | Abclonal | A19707 |
| p-AMPK | CST | 50081 |
| AMPK | CST | 2532s |
| p-LKB1 | CST | 3482 |
| LKB1 | Abclonal | A2122 |
| LKB1 | CST | 3050 |
| LKB1 | CST | 3047 |
| CAMKK2 | proteintech | 11549-1-AP |
| IgG | CST | 2729 |
| Ub | Abcam | ab134953 |
| MKRN1 | Abcam | ab72054 |
| HA | Abclonal | AE036 |
| His | Abclonal | AE068 |
| Flag | sigma | F7425 |
| Flag | CST | 14793 |
| HRP-conjugated Affinipure Goat Anti-Rabbit IgG(H+L) | proteintech | SA00001-2 |
| Goat anti-Rabbit IgG (H+L) Alexa Fluor Plus 488 | invitrogen | A32731 |
| Goat anti-mouse IgG (H+L) Alexa Fluor Plus 647 | invitrogen | A32728 |

**Supplementary Table 4.** Biometric and cardiac parameters of *Myh6^+^* and *Foxm1^fl/fl^Myh6^+^* male mice at 14 days after TAM injection.

| Group | *Myh6^+^* | *Foxm1^fl/fl^Myh6^+^* |
| --- | --- | --- |
| n | 10 | 10 |
| BW (g) | 22.04±0.84 | 20.21±0.78 |
| HR (beats/min) | 493±47 | 510±36 |
| EF (%) | 63.8±8.3 | 26.9±6.4*** |
| FS (%) | 34.2±5.9 | 12.4±3.2*** |
| LVID_d_ | 3.5±0.62 | 4.37±0.50*** |
| LVID_s_ | 2.33±0.56 | 3.83±0.49*** |

Data are expressed as mean ± SD. Data was analyzed using two-way ANOVA followed by Bonferroni post hoc analysis. ***p<0.001 vs. Myh6^+^. BW, body weight; HR, heart rate; EF, ejection fraction; FS, fractional shortening; LVID_d_, left ventricular internal diameter at end-diastole; LVID_s_, left ventricular internal diameter at end-systole.

**Supplementary Table 5.** Biometric and cardiac parameters of *Myh6^+^*, *Foxm1^fl/fl^Myh6^+^*, *Mkrn1^fl/fl^Myh6*^+^ and *Foxm1/ Mkrn1^fl/fl^Myh6^+^* male mice at 14 days after TAM injection.

| Group | *Myh6^+^* | *Foxm1^fl/fl^Myh6^+^* | *Mkrn1^fl/fl^Myh6*^+^ | *Foxm1/ Mkrn1^fl/fl^Myh6^+^* |
| --- | --- | --- | --- | --- |
| n | 10 | 10 | 10 | 10 |
| BW (g) | 22.14±0.73 | 21.74±0.84 | 21.94±0.74 | 22.03±0.57 |
| HR (beats/min) | 493±55 | 512±42 | 508±41 | 499±46 |
| EF (%) | 65.6±6.2 | 24.9±7.6*** | 63.7±5.8 | 39.7±5.3^###^ |
| FS (%) | 35.3±3.9 | 11.1±4.0*** | 33.6±6.0 | 18.93±3.4^###^ |
| LVID_d_ | 3.61±0.56 | 4.46±0.62*** | 3.48±0.55 | 4.13±0.43^##^ |
| LVID_s_ | 2.46±0.42 | 3.73±0.42*** | 2.56±0.40 | 3.4±0.30^##^ |

Data are expressed as mean ± SD. Data was analyzed using two-way ANOVA followed by Bonferroni post hoc analysis. ***p<0.001 vs. *Myh6^+^*; **^###^**p<0.001 vs. *Foxm1^fl/fl^Myh6^+^*. BW, body weight; HR, heart rate; EF, ejection fraction; FS, fractional shortening; LVID_d_, left ventricular internal diameter at end-diastole; LVID_s_, left ventricular internal diameter at end-systole.

**Supplementary Table 6.** Biometric and cardiac parameters of *Myh6^+^* and *Foxm1^TG^Myh6^+^* male mice 4 weeks post-I/R or Sham.

| Group | *Myh6^+^*  Sham | *Foxm1^TG^Myh6^+^*  Sham | *Myh6^+^*  I/R 4weeks | *Foxm1^TG^Myh6^+^*  I/R 4weeks |
| --- | --- | --- | --- | --- |
| n | 10 | 10 | 10 | 10 |
| BW (g) | 21.45±1.04 | 22.11±0.83 | 21.29±1.12 | 20.94±0.94 |
| HR (beats/min) | 483±34 | 502±41 | 514±31 | 493±50 |
| EF (%) | 64.7±5.9 | 62.2±6.2 | 40.0±8.1^***^ | 52.5±3.9**^###^** |
| FS (%) | 34.7±4.2 | 33.1±4.3 | 19.2±4.5^***^ | 26.5±2.5**^###^** |
| LVID_d_ | 3.45±0.28 | 3.78±0.34 | 4.20±0.33^***^ | 3.60±0.28**^###^** |
| LVID_s_ | 2.26±0.29 | 2.36±0.27 | 3.5±0.36^***^ | 2.74±0.30**^###^** |

Data are expressed as mean ± SD. Data was analyzed using two-way ANOVA followed by Bonferroni post hoc analysis. ***p<0.001 vs. Myh6^+^/Sham; **^###^**p<0.001 vs. Myh6^+^/I/R 4weeks. BW, body weight; HR, heart rate; EF, ejection fraction; FS, fractional shortening; LVID_d_, left ventricular internal diameter at end-diastole; LVID_s_, left ventricular internal diameter at end-systole.
